# Pressure Tuning the Jahn–Teller Transition Temperature in NaNiO_2_

**DOI:** 10.1021/acs.inorgchem.1c03345

**Published:** 2022-03-03

**Authors:** Liam A. V. Nagle-Cocco, Craig L. Bull, Christopher J. Ridley, Siân E. Dutton

**Affiliations:** † Cavendish Laboratory, University of Cambridge, JJ Thomson Avenue, Cambridge CB3 0HE, United Kingdom; ‡ ISIS Neutron and Muon Facility, Rutherford Appleton Laboratory, Didcot OX11 0QX, United Kingdom; § School of Chemistry, University of Edinburgh, David Brewster Road, Edinburgh EH9 3FJ, United Kingdom

## Abstract

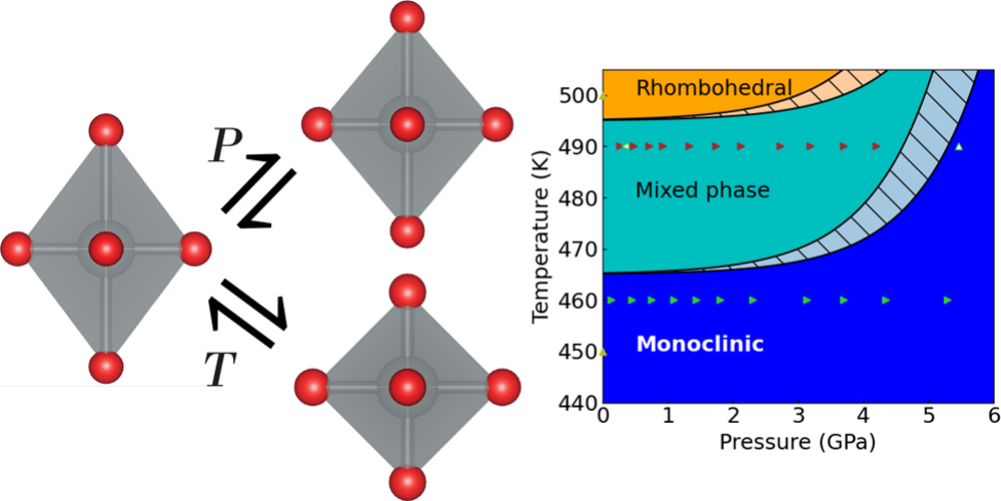

NaNiO_2_ is a layered material consisting of alternating layers of NaO_6_ and Jahn–Teller-active NiO_6_ edge-sharing octahedra. At ambient pressure, it undergoes a broad phase transition from a monoclinic to rhombohedral structure between 465 and 495 K, associated with the loss of long-range orbital ordering. In this work, we present the results of a neutron powder diffraction study on powdered NaNiO_2_ as a function of pressure and temperature from ambient pressure to ∼5 GPa between 290 and 490 K. The 290 and 460 K isothermal compressions remained in the monoclinic phase up to the maximum pressures studied, whereas the 490 K isotherm was mixed-phase throughout. The unit-cell volume was fitted to a second-order Birch–Murnaghan equation of state, where *B* = 119.6(5) GPa at 290 K. We observe at 490 K that the fraction of the Jahn–Teller-distorted phase increases with pressure, from 67.8(6)% at 0.71(2) GPa to 80.2(9)% at 4.20(6) GPa. Using this observation, in conjunction with neutron diffraction measurements at 490 K on removing pressure from 5.46(9)  to 0.342(13) GPa, we show that the Jahn–Teller transition temperature increases with pressure. Our results are used to present a structural pressure–temperature phase diagram for NaNiO_2_. To the best of our knowledge, this is the first diffraction study of the effect of pressure on the Jahn–Teller transition temperature in materials with edge-sharing Jahn–Teller-distorted octahedra and the first variable-pressure study focusing on the Jahn–Teller distortion in a nickelate.

## Abstract 2

In this work, a variable-pressure neutron diffraction study of layered NaNiO_2_ is presented, showing that pressure decreases the magnitude of distortion of Jahn−Teller-distorted Ni^3+^O_6_ octahedra while resulting in an increase in the Jahn−Teller suppression temperature, *T*_JT_.

## 1 Introduction

Many transition metal oxides exhibit a Jahn–Teller (JT) distortion due to degeneracy in the 3d orbitals, manifesting as an elongation or compression of the MO_6_ (M = transition metal ion) octahedra, generally with associated orbital ordering. Previous studies of the effect of pressure on materials containing JT-active ions have found that pressure can entirely suppress the JT distortion and orbital ordering.^1,2^ It has also been observed that application of pressure reduces the magnitude of distortion in MO_6_ octahedra.^3−7^

One well-studied material under pressure is LaMnO_3_ (with JT-active d^4^ Mn^3+^ ions).^2,3,8^ At ambient pressure, it adopts the perovskite structure with corner-sharing MnO_6_ octahedra. An ordered JT distortion results in an orthorhombic symmetry. At ≳750 K, the JT distortion is suppressed, and there is an increase in symmetry first to a cubic phase with octahedral tilting and then at higher temperatures to a rhombohedral phase.^9^ The temperature-driven suppression of the JT distortion coincides with a marked increase in electronic conductivity.^10^ On application of pressure at room temperature *P* < 8 GPa, the JT distortion is decreased through reduction of the long Mn–O bond lengths.^3^ At ∼11 GPa, a rhombohedral phase with no JT distortion coexists with the distorted orthorhombic phase,^2^ becoming single-phase at ∼12 GPa. Similarly, the manganese(III) quadruple perovskite LaMn_7_O_12_ exhibits a complete suppression of the JT distortion at ∼34 GPa.^1^

There are several interesting studies of JT-distorted compounds with edge-sharing octahedra. Here we describe three different examples of classes, all containing JT-active d^4^ Mn^3+^. Mn_3_O_4_, a spinel containing both Mn^3+^ and Mn^2+^, has been found to exhibit different pressure dependence of JT-distorted octahedra depending on morphology; for instance, in single-crystal Mn_3_O_4_, the JT disortion survives to 60 GPa,^11^ whereas there are observed transitions to JT-free phases at much lower pressures in powdered^12^ and nanorod^13^ Mn_3_O_4_. ZnMn_2_O_4_, also with spinel-type structure but with Zn^2+^ on the Mn^2+^ of Mn_3_O_4_, has been studied to a very high pressure (∼52 GPa),^14^ with a transition reported at ∼23 GPa, which has been alternately described as a transition from JT elongation to a slight JT compression^14^ or a spin-crossover transition resulting in an insulator → metal transition.^15^ CuMnO_2_, with a delafossite structure, has also had the pressure dependence of its JT distortion studied.^16^ It exhibits a higher compressibility in the long Mn–O bond than in the short Mn–O bond similar to the case for LaMnO_3_^3^ and other materials^4,5^ up to ∼10 GPa; above this pressure, there is an isostructural phase transition associated with a collapse in the interlayer (*c*-axis) and an increase in the volume of the Mn^3+^O_6_ JT-distorted octahedra.

Nickelates containing JT-active d^7^ Ni^3+^ are far less studied than the manganates under pressure. This may be partly because many materials containing d^7^ Ni^3+^ octahedra do not exhibit a co-operative JT distortion, where the JT distortion is long-range ordered. NdNiO_3_, which has been subjected to a variable-pressure structural study,^17^ is not considered to contain a JT distortion,^18−20^ as is the case for most Ni-containing perovskites.^21^ Similarly, AgNiO_2_ is widely accepted not to contain any kind of JT distortion.^22,23^ LiNiO_2_ is an interesting case as it does not display long-range magnetic or orbital ordering, likely due to Li/Ni site mixing; some experimental results have been interpreted as evidence for a noncooperative JT distortion,^24,25^ although this is debated.^26−28^ Similarly, various nickel-containing perovskites^29^ are subject to discussion regarding whether any kind of JT distortion exists.

NaNiO_2_ is a layered d^7^ nickelate. The presence of the JT distortion in NaNiO_2_ is not subject to debate,^28,30,31^ even among proponents of alternative theories for degeneracy breaking in LiNiO_2_.^28^ NaNiO_2_ is therefore an ideal choice for studying the effect of pressure on the JT distortion in a material that is a nickelate and has edge-sharing octahedra. The room-temperature phase of NaNiO_2_ is a semiconductor, based on its black color and by analogy with LiNiO_2_,^32^ but we do not know of any measurement of the conductivity properties of the high-temperature phase. NaNiO_2_ is of interest because of its magnetic ground state, consisting at ambient pressure of intralayer ferromagnetism and interlayer antiferromagnetism.^33−35^ It has also been studied in recent years because ANiO_2_ (A = alkali metal) is the template compound for Ni-rich alkali metal–transition metal oxides within the field of batteries.^36,37^

**Figure 1:**
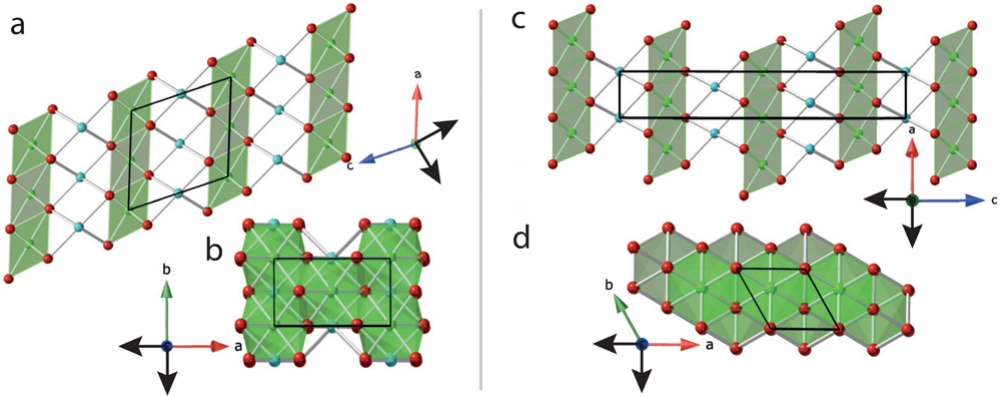
(a and b) Monoclinic, Jahn–Teller-distorted NaNiO_2_ phase along the *b*- and *c*-axes, respectively. (c and d) Rhombohedral, JT-inactive NaNiO_2_ phase along the *b*- and *c*-axes, respectively. Ni^3+^ cations are colored green, O^2–^ anions red, and Na^+^ cations cyan. Na^+^ ions and octahedra are hidden in panels b and d. The solid black quadrilaterals denote the unit cell. The black arrows represent the directions of principal axes of compression projected into the (a and c) *a–c* plane and (b and d) *a–b* plane.

NaNiO_2_ has an ordered JT distortion at room temperature due to degeneracy in e_g_ orbitals in low-spin Ni^3+^. It exhibits a first-order phase transition between 465 and 495 K to an undistorted phase. The crystal structures are shown in Figure 1. The monoclinic (*C*2/*m*) JT-distorted phase consists of alternating layers of edge-sharing NiO_6_ and NaO_6_ octahedra. The NiO_6_ and NaO_6_ octahedra both exhibit angular and bond length distortions from geometrically regular octahedra. Ni, Na, and O ions occupy the 2*a*(0, 0, 0), 2*d*(0, ^1^/_2_, ^1^/_2_), and 4*i*(*x*, 0, *z*) Wyckoff sites, respectively. The rhombohedral (*R*3̅*m*) phase consists of the same arrangement of alternating NiO_6_ and NaO_6_ layers of edge-sharing octahedra, with octahedra bound within layers by O_4_ tetrahedra. In this phase, Ni, Na, and O ions occupy the 3*b*(0, 0, ^1^/_2_), 3*a*(0, 0, 0), and 6*c*(0, 0, *z*) Wyckoff sites, respectively. The unit cell remains centrosymmetric, with the change in symmetry due solely to the reduction in magnitude of the JT distortion and the resulting nonvariable M–O (M = Na or Ni) bond lengths.

In this work, we present a structural study of NaNiO_2_ as a function of temperature between 290 and 500 K and pressures up to 5.46(9) GPa. We demonstrate using the 490 K isotherm that the JT transition temperature increases between 2 and 4.2 GPa, increasing more rapidly with pressure at higher pressures, while the degree of distortion decreases over this pressure range.

## 2 Methods

### 2.1 Sample Preparation and Characterization

Samples were prepared by solid state synthesis. Na_2_O_2_ (Alfa Aesar, 95%) and NiO (Alfa Aesar, 99.995%) were mixed and pelletized in a 1.05:1 Na:Ni molar ratio, with excess Na to account for Na loss during heating. The sample was heated to 973 K for 70 h in a tube furnace under a constant flow of O_2_. To prevent reaction with moisture, the sample was stored and handled in an inert Ar atmosphere. X-ray diffraction (XRD) data were obtained using a Bruker D8 Discover powder (Cu Kα_1,2_; λ = 1.541 Å) diffractometer. A Mira3 TESCAN scanning electron microscope was used to obtain SEM images of the morphology of NaNiO_2_, with an accelerating electron voltage of 3 kV (for SEM images, see the Supporting Information).

### 2.2 Ambient-Pressure Neutron Diffraction

Ambient-pressure neutron diffraction was performed using the NOMAD instrument^38^ at the Spallation Neutron Source of Oak Ridge National Laboratory (Oak Ridge, TN). NaNiO_2_ was sealed in a glass ampule for the measurements. Heating was performed using a furnace. The sample was measured during heating at 293, 450, and 500 K and after cooling at 316 K.

### 2.3 Variable-Pressure Neutron Diffraction

Variable-temperature and -pressure neutron diffraction studies were performed at the PEARL instrument,^39^ ISIS Neutron and Muon Source, UK, using a V3 Paris-Edinburgh press. The sample was measured between 0.107(8)  and 4.24(5) GPa at 290 K, 0.130(10)  and 5.29(8) GPa at 460 K, and 0.254(17)  and 4.20(6) GPa at 490 K. NaNiO_2_ was packed into an encapsulated null scattering TiZr gasket that was loaded in a zirconia-toughened alumina toroidal profile anvil, with a lead pellet for pressure calibration.^40^ An anhydrous deuterated methanol/ethanol mixture (4:1 by volume) was used as a pressure-transmitting medium for the ambient-temperature isothermal compression experiment. Preliminary measurements indicated that NaNiO_2_ reacted with the methanol/ethanol solution at higher temperatures (Figure S2), so a FC77/FC84 fluorinert mixture (1:1 volume) (purchased from 3M) was used for the 460 and 490 K isotherms. The data were processed and corrected using Mantid.^41^

### 2.4 Diffraction Analysis

Diffraction data were analyzed using the software package topas 5,^42^ utilizing Pawley fitting^43^ and Rietveld refinement.^44^ For NaNiO_2_, preliminary analysis of NOMAD data indicated Na occupancy was 1 within error; hence, the site occupancy of all sites during all further refinement was fixed at 1. Thermal *B*_eq_ parameters were allowed to refine but constrained to be positive and not exceed a value of 5 Å^2^. All atomic positions were refined within symmetry constraints. The background was fitted by a Chebyschev polynomial (order 6 for PEARL data, order 11 for NOMAD data, and order 19 for XRD data). For XRD data, a TCHZ peak shape was used.^45^ Peak shapes used for neutron data are discussed in section II of the Supporting Information. For PEARL, only the 90° detection bank was used, but for NOMAD, a combined refinement was performed using banks 2–5 (2θ = 31°, 67°, 122°, and 154°, respectively).

## 3 Results

### 3.1 Ambient-Pressure Structural Properties

Powder X-ray diffraction of the as-synthesized NaNiO_2_ indicated the formation of a phase-pure product. SEM of the material indicates the sample is polycrystalline with particulates between 0.2 and 5 μm in diameter (Figure S13). Rietveld refinement using the reported monoclinic *C*2/*m* space group (Figure S1 and Table S1) yielded lattice parameters consistent with prior reports.^30,46^

**Figure 2:**
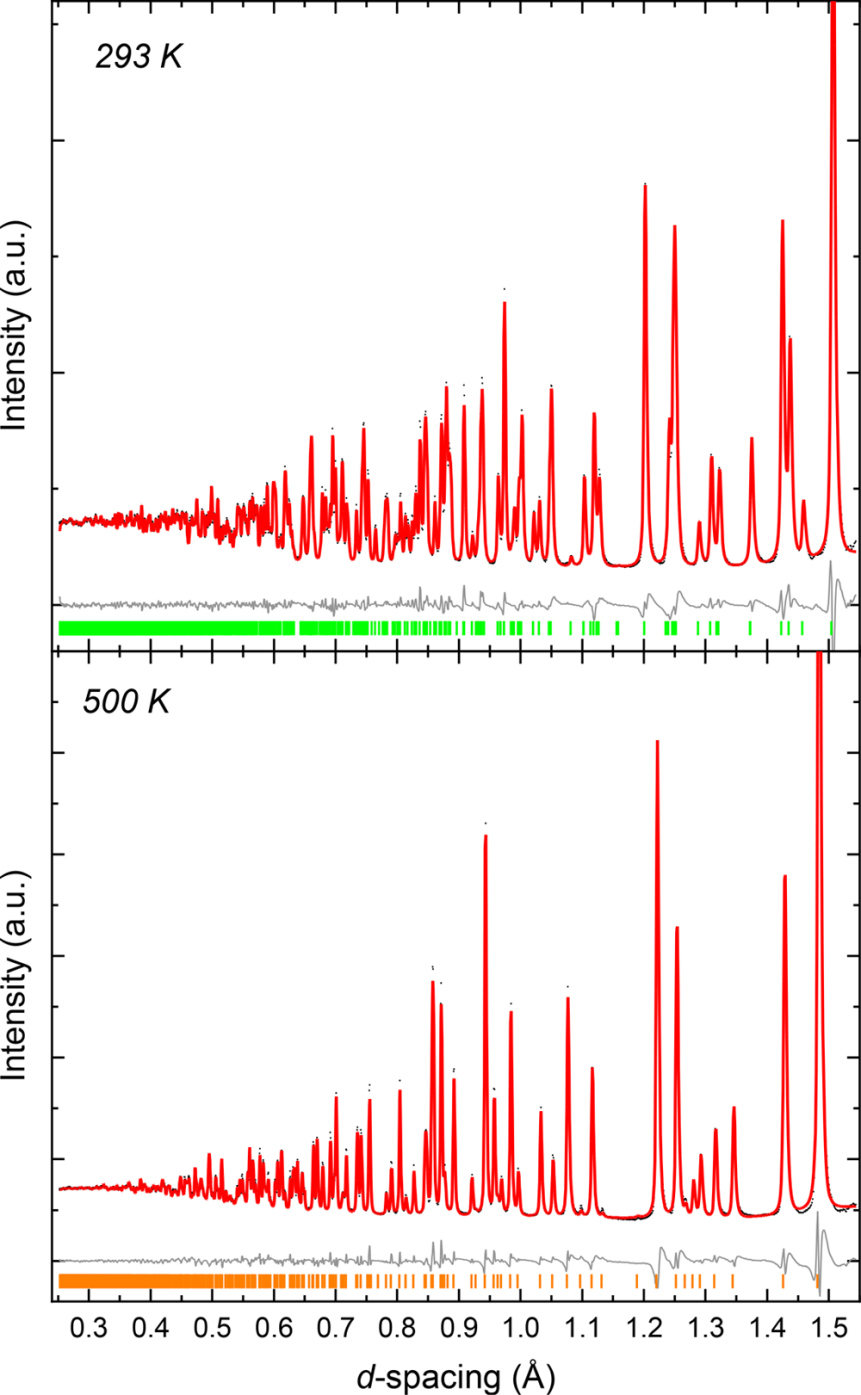
Rietveld refinements for the ambient-pressure, variable-temperature neutron diffraction measurements of NaNiO_2_ on bank 5 of NOMAD (2θ = 154°) at 293 K (top) and 500 K (bottom). Black dots for measured data, red line for the calculated diffraction pattern from Rietveld refinement, and gray line for *Y*_obs_ – *Y*_calc_. Green and orange tick marks show expected reflections for the monoclinic and rhombohedral phases, respectively.

The reported monoclinic → rhombohedral phase transition in NaNiO_2_ was investigated using neutron powder diffraction at ambient pressure on the NOMAD instrument. Rietveld refinement (Figure 2) shows the phase transition occurs between 450 and 500 K and is reversible on cooling. The lattice parameters (Table S2) all exhibit positive thermal expansion and are consistent with previous measurements.^30,31^

In the monoclinic structure, the NiO_6_ octahedra exhibit a cooperative JT distortion with two longer Ni–O bonds, whereas in the high-temperature rhombohedral phase, all six Ni–O bonds are equivalent (Figure 1). The degree of bond length distortion within individual NaO_6_ and NiO_6_ octahedra can be evaluated using a number of distortion metrics, calculated using topas 5.^42^ Here we consider the effective coordination number^47^ and the bond length distortion indices,^48^ which measure distortion in octahedra by quantifying the difference from the average value of the distances between the central cation and the coordinated oxygen anions. The general form of the effective coordination number, ECoN, and bond length distortion index, *D*, is given in the Supporting Information. The equations as applicable to monoclinic NaNiO_2_ are
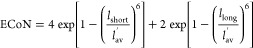
1where *l*_av_^′^ is a modified average bond length defined in the Supporting Information and
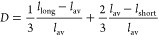
2where *l*_av_ is the average bond length and *l*_long_ and *l*_short_ are the long and short bond lengths, respectively.

In the rhombohedral structure, the effective coordination number and bond length distortion index of the NiO_6_ and NaO_6_ octahedra are constrained by symmetry to values of 6 and 0, respectively. In the monoclinic structure, departure from these values indicates bond length disproportionation and is primarily attributable to the JT distortion. These changes are significantly larger for the JT-active NiO_6_ octahedra than for the NaO_6_ octahedra.

Throughout the measurement, the bond length distortion index{effective coordination} of NaO_6_ octahedra remains very near its high-symmetry value of 0{6}; for example, at 293 K, the value of the bond length distortion index{effective coordination} in NaO_6_ octahedra is 0.00581(11){5.99232(19)}, compared with 0.05463(14){5.309(3)} in NiO_6_ octahedra. This is indicative of much greater distortion in bond lengths for NiO_6_ octahedra, consistent with the JT distortion. The values of the bond length distortion index are on the same order of magnitude as recent studies of JT-distorted Mn^3+^O_6_-containing compounds.^16,49^

Inconsistency in bond length is not the only distortion of the octahedra from regular octahedra. A regular octahedron would have bond angles θ_O–M–O_ of 90° for nearest-neighbor O anions. However, in the JT-active monoclinic phase and the JT-inactive rhombohedral phase, there is variance from this ideal bond angle. Non-nearest-neighbor oxygen anions are constrained to have 180° bond angles via the central cation, so the 12 bond angles in an octahedron are each paired with another O–M–O bond, with the paired bond angles sharing one oxygen in common and with their nonshared oxygen anions occurring along a straight line through the central cation (for a visual representation, see Figure S14). We define these bond angles as θ_O–M–O_ = 90° ± Δ, where the two angles in a pair have opposite signs preceding the Δ. Δ can also be considered a measure of the extent of angular distortion. In the rhombohedral structure, there is only one value of Δ for each type of octahedron, with half of the O–M–O bond angles being 90° + Δ and the other half being 90° – Δ. In the monoclinic unit cell where octahedra have two long M–O (M = Na or Ni) bonds and four short M–O bonds, there are four nearest-neighbor bond angles between short and short bonds and eight nearest-neighbor bond angles between short and long bonds. We therefore must define two values of Δ for the bond angles in the monoclinic phase, Δ_short–short_ and Δ_long–short_, respectively. Table 1 shows these values of Δ at each temperature. It is clear that NaO_6_ octahedra exhibit far greater bond angle distortion than NiO_6_ octahedra, in contrast to the bond length distortion that is greater for NiO_6_ octahedra. This is not unexpected, given that crystal field effects will result in much greater stability for open-shell d^7^ Ni^3+^ in an octahedral configuration, minimizing bond angle variance, whereas this will not be a factor for closed-shell Na^+^ cations.

**Table 1:** Values of Δ for Bond Angles θ_O–M–O_ (M = Na or Ni; θ_O–M–O_= 90° ± Δ) as a Function of Temperature^a^

		NiO_6_ (deg)		NaO_6_ (deg)	
phase	*T* (K)	Δ_short–short_^Ni^	Δ_long–short_^Ni^	Δ_short–short_^Na^	Δ_long–short_^Na^
*C*2/*m*	293 (−)	6.134(17)	5.456(19)	14.494(12)	9.708(14)
*C*2/*m*	450 (*↑*)	6.163(19)	5.50(2)	14.564(14)	9.844(16)
*R*3̅*m*	500 (*↑*)	6.135(15)		11.777(13)	
*C*2/*m*	316 (*↓*)	6.121(18)	5.46(2)	14.505(13)	9.731(15)

a For definitions, see the text. The arrow next to the temperature indicates whether the data were collected on warming or cooling of the sample.

### 3.2 Variable-Pressure Neutron Diffraction

**Figure 3:**
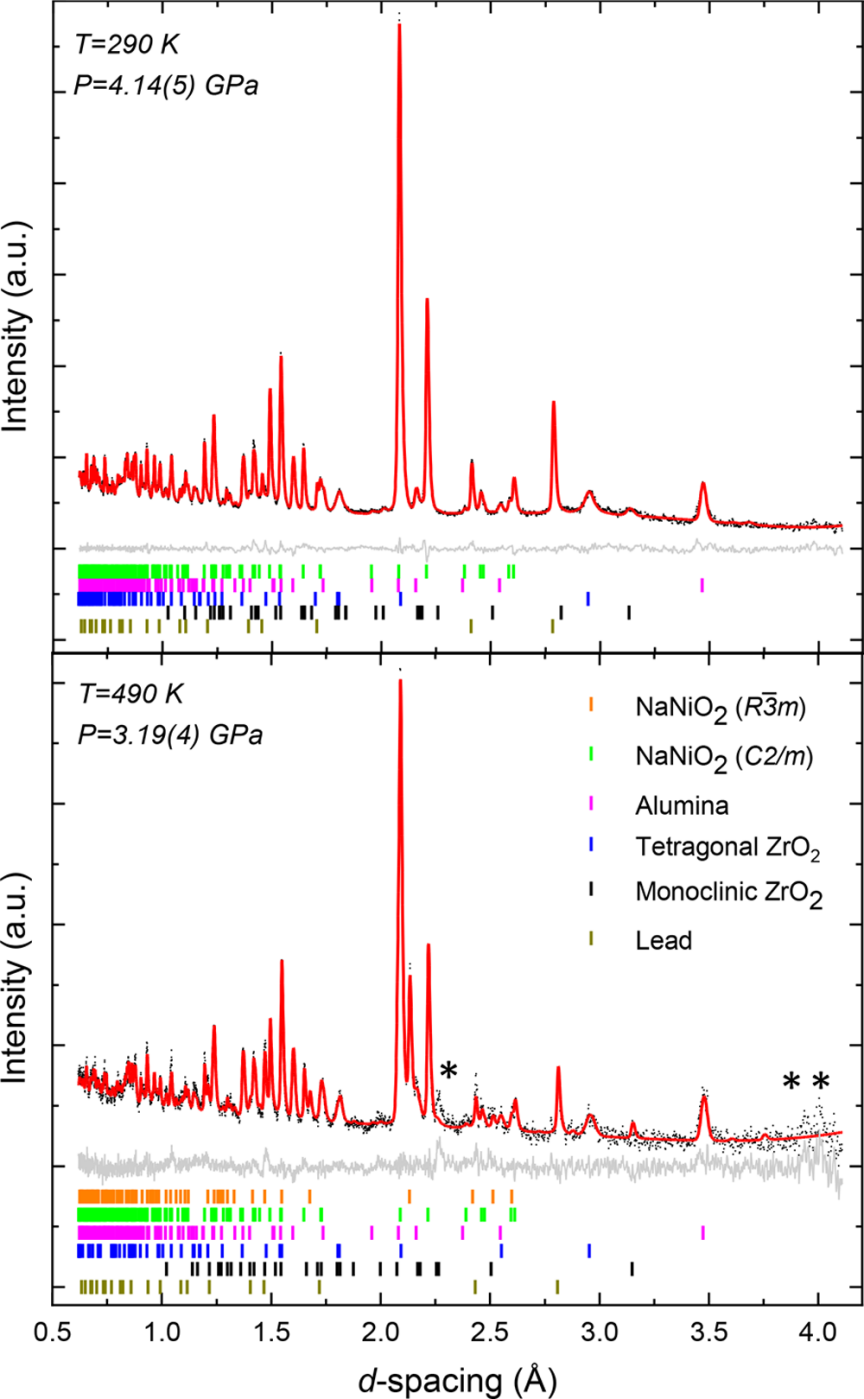
Rietveld refinements for the variable-pressure neutron diffraction data of NaNiO_2_. Representative plot with monoclinic NaNiO_2_ only (top) and a representative plot with both monoclinic and rhombohedral NaNiO_2_ (bottom). Black dots for measured data, red line for the calculated diffraction pattern from Rietveld refinement, and gray line for *Y*_obs_ – *Y*_calc_. Unfitted peaks are marked with an asterisk and arise from crystalline fluorinert (Figure S3).

The effect of pressure on the JT distortion in NaNiO_2_ was explored at 290, 460, and 490 K, with an example Rietveld refinement shown in Figure 3. Over the entire pressure and temperature range studied, NaNiO_2_ could be described using the previously reported ambient-pressure crystal structures. Diffraction data also included contributions from alumina and zirconia in the sample environment and the lead used to determine the applied pressure; these are also included in the structural refinements. In addition, at higher temperatures (460 and 490 K) and pressures, additional peaks attributed to crystallization of the fluorinert pressure media (Figure S3) are observed in the measurements.

**Figure 4:**
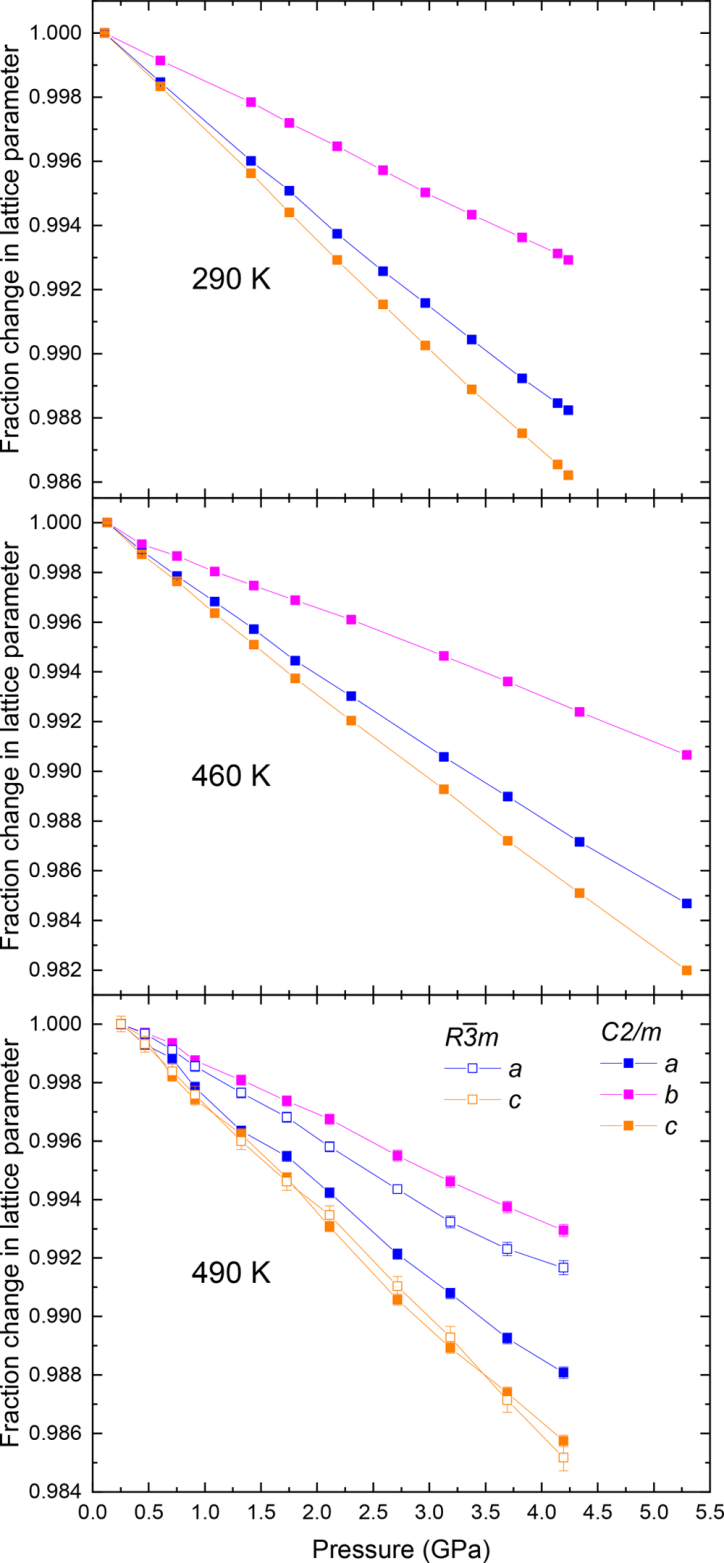
Fractional contraction of lattice parameters obtained by Rietveld refinement^44^ as a function of temperature and pressure for the monoclinic *C*2/*m* and rhombohedral *R*3̅*m* phases of NaNiO_2_. Where error bars are not visible, it is because they are smaller than the data point. Lines are a guide for the eye.

Rietveld analysis (Figure 3) shows that NaNiO_2_ remained in the monoclinic phase at 290 K [up to 4.24(5) GPa] and 460 K [up to 5.29(8) GPa]. However, the measurements at 490 K capture NaNiO_2_ midway through its transition from JT-distorted *C*2/*m* monoclinic to JT-inactive *R*3̅*m* rhombohedral, and throughout this isotherm, the NaNiO_2_ is mixed-phase.

The lattice parameters (Figure 4) show the expected variation with temperature and pressure. The rhombohedral and monoclinic phases have similar compressibility, and in both, NaNiO_2_ is considerably more compressible in the interlayer direction (*c*-axis) than in the intralayer (*a–b*) plane. With reference to Figure 1, we note that compression within the plane results in changes to the highly ionic Na^+^–Na^+^ interactions and the less ionic but still repulsive Ni^3+^–Ni^3+^ interactions, whereas compression in the interlayer direction will compress the Ni–O and Na–O bonds that are softer due to the nearest-neighbor interaction lacking a Coulomb repulsive force. This higher compressibility in the interlayer direction is consistent with that seen in another material with alternating layers of edge-sharing octahedra, the honeycomb iridate Na_2_IrO_3_.^50,51^

Within the plane, in monoclinic NaNiO_2_, the *b*-axis is less compressible than the *a*-axis. A reason for this might be that Na^+^–Na^+^ and Ni^3+^–Ni^3+^ interactions are parallel to the direction of compression for the *b*-axis, maximizing the increase in Coulomb repulsion with decreasing the lattice parameter due to compression, whereas there are no Na^+^–Na^+^/Ni^3+^–Ni^3+^ interactions with components only along the *a*-axis. Another contribution may be that the Na^+^–Na^+^ and Ni^3+^–Ni^3+^ ionic distances parallel to the *b*-axis are considerably shorter than the distances that can be projected onto the *a*-axis [∼2.85 and ∼3.02 Å, respectively, at 290 K and 0.107(8) GPa].

**Figure 5:**
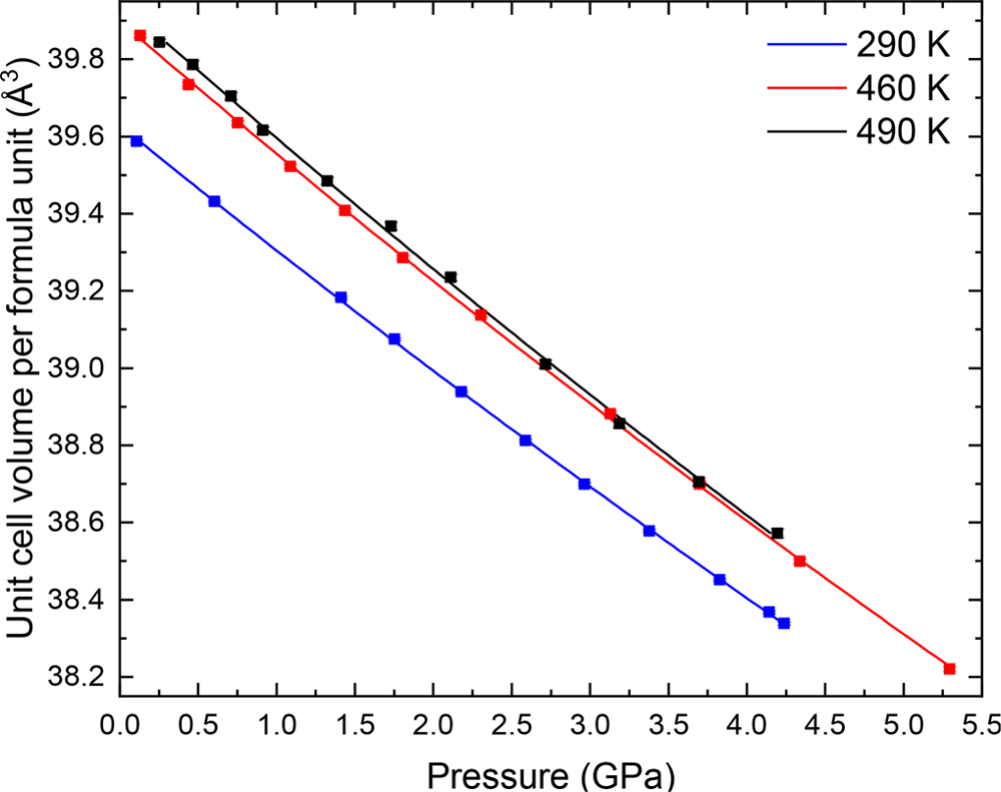
Variation in unit-cell volume per formula unit for the monoclinic *C*2/*m* phase. Solid data points show experimentally derived values, and the solid line shows the determined second-order Birch–Murnaghan equation of state. Full lattice parameters are listed in Tables S3–S5. Where error bars are not visible, it is because they are smaller than the data points.

PASCal^52^ was used to obtain the bulk modulus for each isotherm, using a second-order Birch–Murnaghan equation of state.^53^ A plot of the unit-cell volume obtained by Rietveld refinement, as a function of pressure, with a fit of this equation of state, is shown in Figure 5 and listed in Table 2. For the monoclinic phase, , which is consistent with a structure with positive thermal expansion. *B* decreases with an increase in temperature, meaning that compressibility increases with temperature. At 290 K, *B* is 119.6(5) GPa. This is comparable with a similar JT-distorted material with edge-sharing octahedra, CuMnO_2_, which has a bulk modulus of 116(2) GPa.^16^ It is, however, substantially less than the reported bulk modulus for ZnMn_2_O_4_ of 197(5) GPa,^14^ and although there are several different reported values for Mn_3_O_4_ depending on the phase and morphology,^11−13^ all are higher than what we report for NaNiO_2_. LaMnO_3_ is not entirely comparable owing to the LaO_12_ units and corner-sharing octahedra, but for reference, it has a reported bulk modulus of 108(2) GPa.^3^

**Table 2:** Parameters Determined from the Second-Order Birch–Murnaghan Equation of State, Obtained Using PASCal^52^ (section V of the Supporting Information and Table S12)

phase	temperature (K)	*V*_0_ (Å^3^)	*B* (GPa)
*R*3̅*m*	490	119.83(2)	113(1)
*C*2/*m*	490	79.900(16)	110(1)
	460	79.798(9)	113.5(6)
	290	79.258(7)	119.6(5)

The directions of the principal axes of compression are determined using PASCal^52^ (Figure S13). These are the axes in which compression occurs linearly with pressure and do not necessarily align with the crystallographic axes in crystalline materials. The principal axis directions projected onto the *a–b* plane do not change between the monoclinic and rhombohedral phases. However, the interlayer direction is a principal axis for the rhombohedral phase, but not for the monoclinic phase where two principal axes are at an angle to the interlayer direction (Figure 1). Interestingly, the axis of JT elongation does not correspond to any of the principal axes (Table S11). There is some temperature dependence in the principal axis directions, likely owing to differing temperature-dependence between the lattice parameters (Tables S3–S5). The compressibility of NaNiO_2_ in each of the principal axes is consistent in magnitude with the relative variation in lattice parameters with pressure (Table S11).

We now consider the pressure dependence of the bond length distortion index and effective coordination (Figure S8 and Tables S8–S10). As in the ambient-pressure measurements, the bond length distortions are significantly larger in the NiO_6_ than in the NaO_6_ octahedra, with the most significant variation being the increase in effective coordination [5.387(10) at 0.107(8) GPa to 5.504(13) at 4.24(5) GPa at 290 K] and the decrease in the bond length distortion index [from 0.0512(5) to 0.0458(7) in the same pressure range at 290 K] of the NiO_6_ octahedra in the monoclinic phase on application of pressure. NaO_6_ octahedra exhibit far smaller changes in the bond length distortion index and effective coordination, with the overall behavior not seeming to exhibit a consistent change with pressure; effective coordination remains between 5.98 and 5.99 throughout the 290 K isotherm.

**Figure 6:**
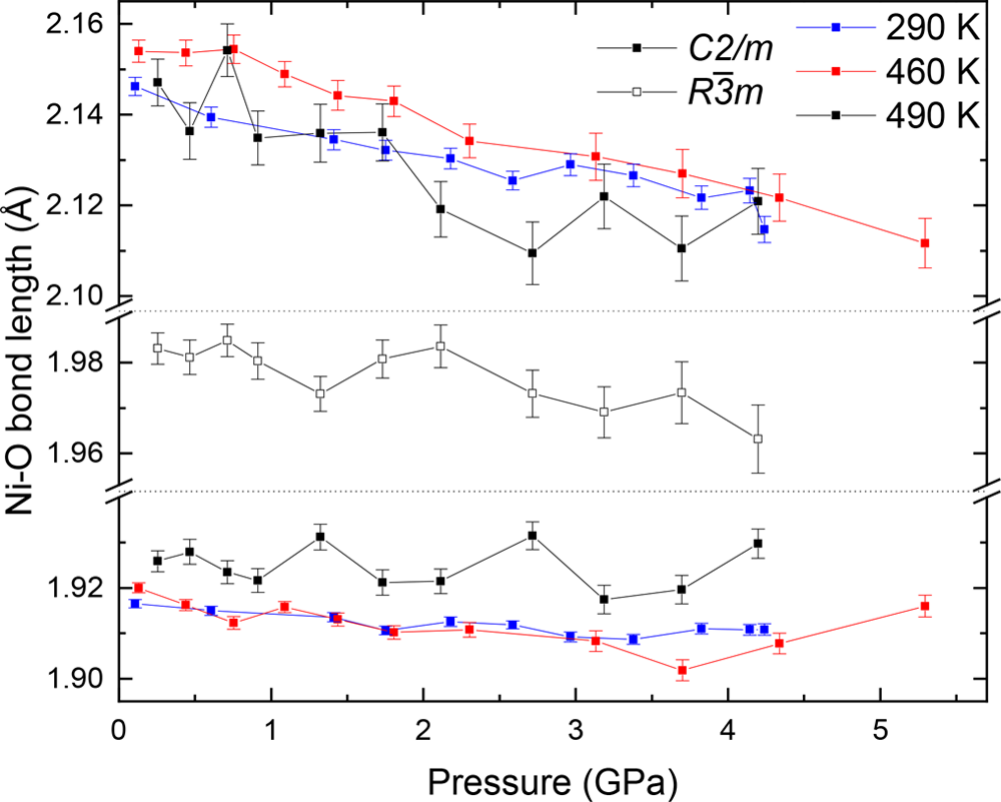
Ni–O bond lengths, as a function of pressure, and associated error of monoclinic NaNiO_2_ at 290, 460, and 490 K, with the JT-inactive rhombohedral-phase bond lengths shown for 490 K. Lines are a guide for the eye.

The differing behavior of the bond length distortion index between NiO_6_ and NaO_6_ octahedra is likely attributable to the fact that NiO_6_ is JT-active and NaO_6_ is not and suggests that the pressure is decreasing the magnitude of JT distortion. We investigate this by considering the direct manifestation of the JT effect in NaNiO_2_. The Ni–O bond lengths of the monoclinic and rhombohedral phases as a function of pressure are shown in Figure 6. The short Ni–O bonds are less sensitive to the effect of pressure than the long Ni–O bonds, indicating that the difference between long and short Ni–O bond lengths is decreasing with pressure. We also observe that the average monoclinic bond length is consistently larger than the rhombohedral bond length at 490 K (Figure S9). In the NaO_6_ octahedra (Figure S5), there is an approximately linear variation of the Na–O bond lengths with pressure. We conclude that the anisotropy of Ni–O bond compression is a consequence of the JT distortion in NiO_6_ octahedra.

The observed decrease in difference between long and short Ni–O bonds with pressure is also reported for other materials containing a JT distortion, such as LaMnO_3_,^3^ KCuF_3_,^4^ and CuAs_2_O_4_.^5^ This is equivalent to the observed tendency with pressure of NiO_6_ octahedra bond length distortion index and effective coordination toward their symmetry-constrained values of 0 and 6, respectively. It indicates that the symmetry of JT-distorted octahedra increases with application of pressure in monoclinic NaNiO_2_, consistent with prior reports.^3−7^

A previous study of LaMnO_3_ attempted to extrapolate a linear fit to the pressure dependence of JT-distorted bond length and estimated a critical JT suppression pressure, *P*_JT_, of ∼18 GPa.^3^ Such an extrapolation could be performed for NaNiO_2_ yielding a *P*_JT_ of ∼50 GPa, converging at a Ni–O bond length of 1.85 Å at 290 K. However, this value is unlikely to be representative of the true *P*_JT_ of the JT distortion in NaNiO_2_. A later study of LaMnO_3_ found that the JT distortion was suppressed at a lower pressure of ∼12 GPa, suggesting such extrapolation does not yield accurate predictions.^2^ In addition, studies of other JT-distorted materials such as [(CH_3_)_2_NH_2_][Cu(HCOO)_3_]^6^ and CuMnO_2_^16^ have found that this pressure dependence of the JT-disproportionated bond length exists only up to a certain pressure, beyond which there is a change in behavior, which renders such extrapolation of low-pressure behavior meaningless.

**Figure 7:**
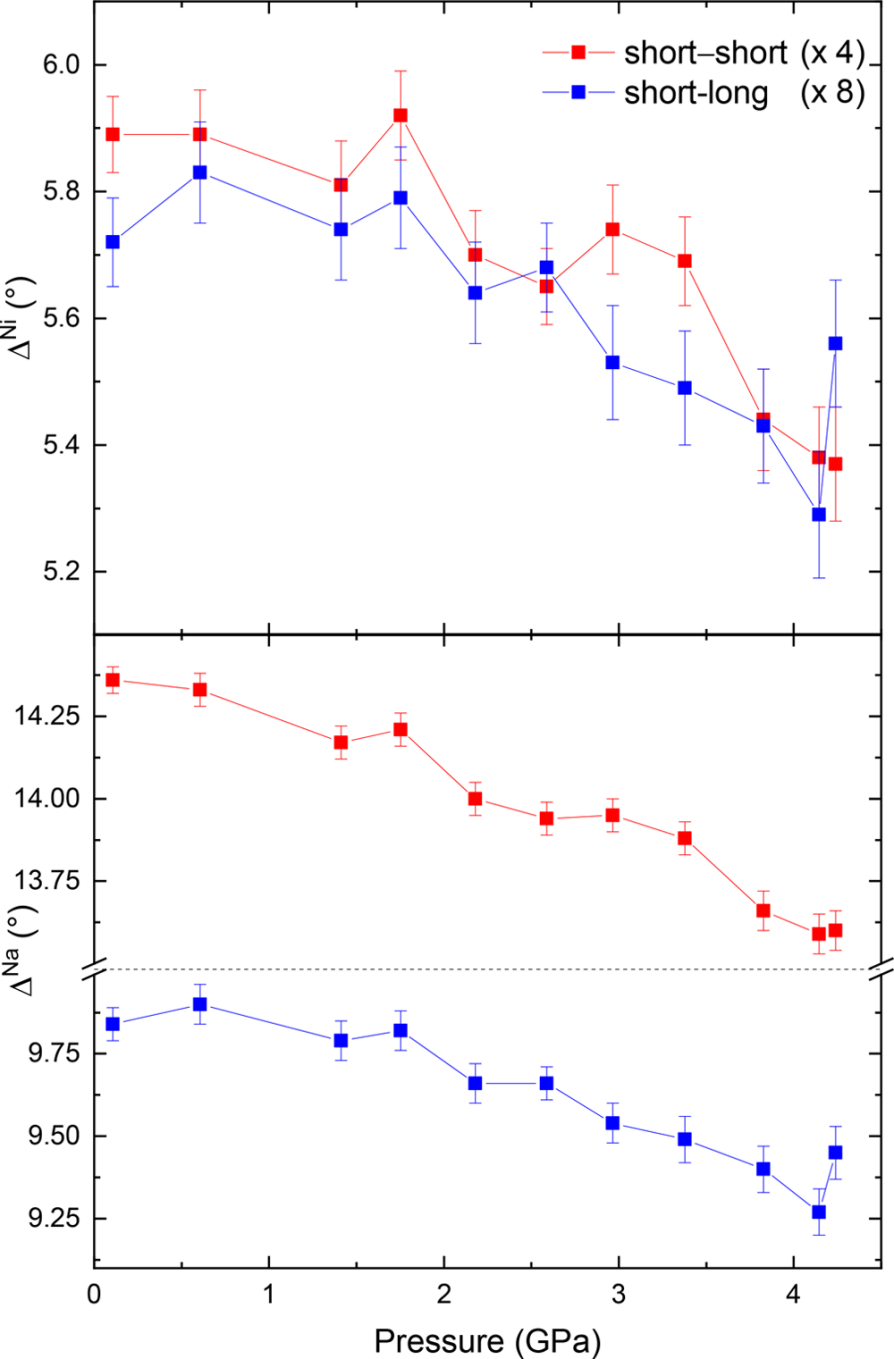
Values of Δ for NiO_6_ and NaO_6_ octahedra as a function of pressure at 290 K, representing the magnitude of angular distortion as nearest-neighbor bond angles take the value 90° ± Δ. The two different Δ values in monoclinic NaNiO_2_ are between two short bonds (red) and between a short and long bond (blue), where bonds are short or long due to the JT distortion. Lines are a guide for the eye.

We earlier defined the bond angles θ_short–short_^M^ and θ_long–short_^M^ (M = Na or Ni) for monoclinic NaNiO_2_, and the associated Δ values that reduce the number of parameters needed to describe the behavior. We plot these Δ values in Figure 7 for the 290 K isotherm. These plots show that throughout the studied pressure range, the degree of angular distortion is far greater for NaO_6_ than for NiO_6_, as was the case at ambient pressure (Table 1). We can also see that with application of pressure, Δ is decreasing; this indicates increasing symmetry toward the 90° bond angle for a perfect octahedron, analogous to the increasing symmetry with pressure we see with the bond length distortion index.

The pressure dependence of the NaO_6_ and NiO_6_ octahedral volume in NaNiO_2_ (Figure S7) shows that the changes in volume display different pressure dependence for NiO_6_ octahedra and NaO_6_ octahedra, as compared with the unit cell. The relative compressibility of NaO_6_ octahedra is higher than that of the entire unit cell, and NiO_6_ octahedra are much more resistant to compression. It has been shown that for perovskites with AO_12_ and BO_6_ polyhedra the parameters *M*_A_ and *M*_B_ can be used to predict the relative compressibility of the polyhedra via the equation β_B_/β_A_ = *M*_A_/*M*_B_, in which  is the bond compressibility, *R*_*i*_ is the distance between the central cation and the *i*th O anion, and *M*_*i*_ is a bond-valence parameter defined in the Supporting Information.^54^ We apply this model to NaNiO_2_ and find that *M*_Ni_ > *M*_Na_ throughout the 290 K isotherm (Figure S12). Accounting for the different values of *R*_*i*_, this indicates that , which is consistent with our observation that NaO_6_ octahedra are more compressible than NiO_6_ octahedra. This may be due to differences in the electronic configuration for closed-shell Na^+^ and open-shell Ni^3+^, or Na^+^ being a much larger ion than Ni^3+^.

We now consider a related model proposed by Angel et al., again in the context of perovskites,^55^ whereby a transition temperature *T*_c_ associated with an octahedral phase transition will exhibit d*T*_c_/d*P* < 0 if octahedra are more compressible than the extra-framework cation sites (analogous to the NaO_6_ octahedra in NaNiO_2_) and d*T*_c_/d*P* > 0 if octahedra are less compressible. Our structural analysis shows the enhanced compressibility of NaO_6_ octahedra compared to that of NiO_6_, so this model predicts the observed increase in *T*_JT_ with pressure. It is worth noting that there are more degrees of freedom in the layered NaNiO_2_ structure so the relationships between distortions in NiO_6_ and NaO_6_ may not be as strongly coupled as in the perovskites. However, the basic hypothesis of the model of Angel et al. appears to be applicable to NaNiO_2_.

**Figure 8:**
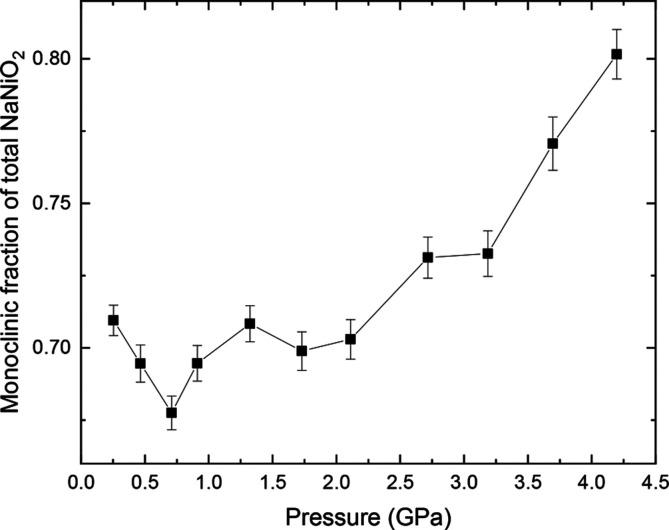
Fraction of NaNiO_2_ that is in the monoclinic phase at 490 K, as a function of pressure.

Along the 490 K isotherm, both monoclinic and rhombohedral NaNiO_2_ were observed to coexist. The fraction of NaNiO_2_ in the low-temperature, JT-distorted monoclinic phase is shown in Figure 8. The fraction remains approximately stagnant to ∼2 GPa, beyond which it consistently increases with pressure. In the range where it is increasing, the monoclinic fraction at 490 K increases from 67.8(6)% at 0.71(2) GPa to 80.2(9)% at 4.20(6) GPa. This indicates that *T*_JT_ increases with an increase in pressure beyond ∼2 GPa, consistent with our prediction based on octahedral compressibility.

To explore the *P*–*T* dependence of the transition, the sample was heated at 5.29(8) GPa from 460 to 490 K after measuring the variable-pressure 460 K isotherm. At ambient pressure, this would result in a mixed monoclinic/rhombohedral phase. However, at the resulting high pressure of 5.46(9) GPa, we did not observe the emergence of any rhombohedral peaks in the diffraction pattern. A subsequent decrease in the pressure to 0.342(13) GPa at the same temperature, 490 K, did yield the emergence of rhombohedral peaks (Figure S4), further supporting our interpretation that *T*_JT_ is increasing with pressure.

## 4 Discussion

**Figure 9:**
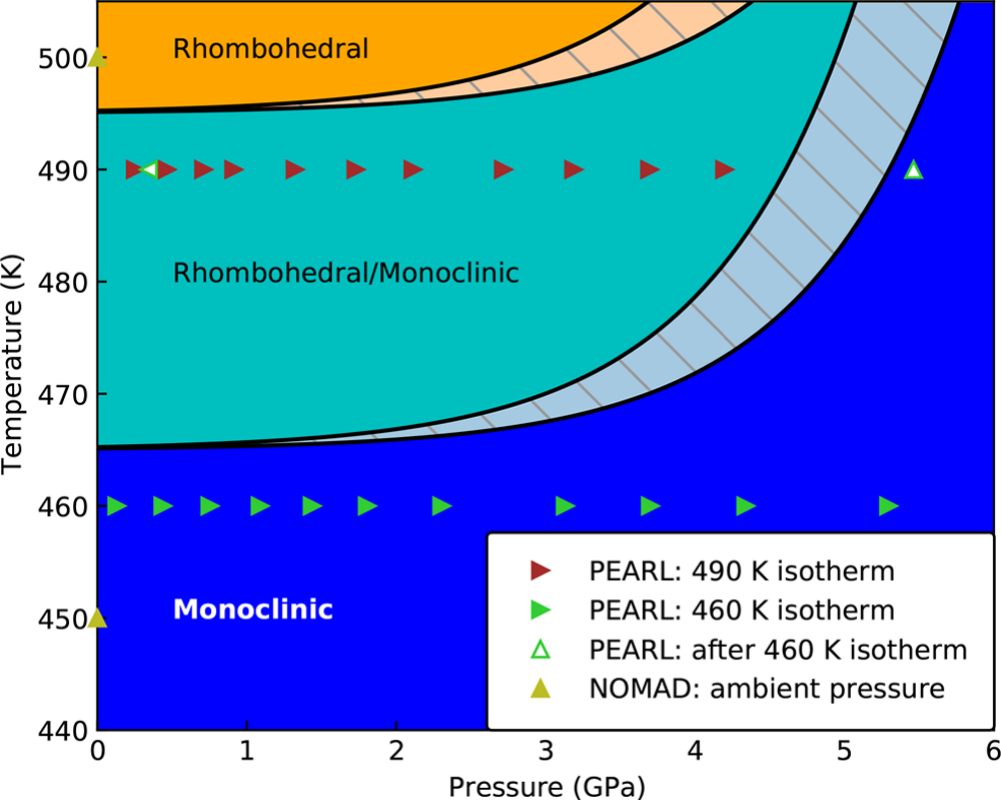
Tentative phase diagram showing the structure of NaNiO_2_ as a function of pressure and temperature. Triangles denote diffraction measurements and point left or right if *P* was decreasing or increasing or up or down if *T* was increasing or decreasing, respectively. The precise boundaries of the three regions are estimates based on available data, with the results in refs (31), (46), used to estimate the broadness of the transition.

The results of our *P*–*T* study on NaNiO_2_ are summarized in a phase diagram (Figure 9). To the best of our knowledge, this is the first study on the effect of pressure on the JT transition temperature in a material containing edge-sharing MO_6_ octahedra and the first variable-pressure study focusing on the JT distortion in a nickelate. Comparison between the results of this study and previous works must therefore rely on the work done on non-nickelate materials.

Like the perovskite materials LaMnO_3_^3,8^ and KCuF_3_,^4^ NaNiO_2_ exhibits far greater compressibility in the JT-elongated O–Ni–O axis than in the JT-compressed O–Ni–O axes, with the JT distortion in both NaNiO_2_ and the previously discussed perovskites decreasing in magnitude with pressure. The consistent behavior with other JT-active materials is also clear evidence that the charge disproportionation model proposed for LiNiO_2_^26−28^ and some Ni^3+^-containing perovskites^21^ is not applicable to NaNiO_2_.

A novel behavior we observe in NaNiO_2_ is that *T*_JT_ increases with application of pressure. For comparison, in LaMnO_3_, the JT distortion is suppressed at ∼12 GPa, indicating that *T*_JT_ is decreased to room temperature from ∼750 K by 12 GPa.^2^ This mechanism seems unlikely in NaNiO_2_ due to the increasing *T*_JT_ with pressure, although we cannot exclude the possibility that a reversal above our maximum measured pressure may result in a decrease in *T*_JT_. Additionally, there is a trend observed in this and other works;^3−7^ the magnitude of distortion due to the JT effect decreases with pressure. This could be interpreted as meaning that there is some pressure where the distortion is entirely suppressed and the NiO_6_ octahedra achieve a bond length distortion index of zero, consistent with the absence of an ordered JT distortion. However, this is at odds with recent reports^6,16^ that show that at some pressures the long and short bonds in JT-distorted octahedra eventually stabilize at different lengths. It is therefore not clear how exactly the JT distortion is suppressed in NaNiO_2_ with high pressure, and further investigation is needed to elucidate this.

We should once again note that the conductivity behavior of the high-temperature phase of NaNiO_2_ also remains unexplored. There is a significant decrease in resistivity with JT suppression in LaMnO_3_,^10^ and there is a possibility for similar behavior in high-temperature rhombohedral NaNiO_2_. Density functional theory calculations on rhombohedral, JT-free LiNiO_2_ (which is isostructural with the high-temperature phase of NaNiO_2_) have suggested metallic behavior.^56^ On a similar note, a broad first-order transition between two structures with a group–subgroup relationship in SrCrO_3_^57^ featured the coexistence of electronic phases. If the high-temperature phase of NaNiO_2_ were indeed metallic, this metallic behavior could explain why d*T*_JT_/d*P* > 0 in this material, as application of pressure may result in narrowing of Ni(3d)–O(2p) bands, pushing the metal-to-insulator phase transition to higher and higher temperatures, and electronic-phase coexistence could provide an explanation for the very broad nature of the transition.

## 5 Conclusion

The key finding of this study is that in NaNiO_2_, *T*_JT_ increases slightly with application of pressure while JT-distorted NiO_6_ octahedra become more symmetric, as demonstrated by the pressure dependence of two distortion metrics (effective coordination and bond length distortion index). While the latter is a well-documented property of JT-distorted materials, the former is in contrast to the JT distortion in LaMnO_3_.^2^ NaNiO_2_ is more resistant to pressure than other similar materials, having a higher bulk modulus [*B* = 119.6(5) GPa at 290 K] than similar perovskites,^3,4^ Prussian Blue analogues,^7^ and layered honeycomb structures,^50^ although its bulk modulus is very similar to that of JT-distorted edge-sharing CuMnO_2_^16^ and is less than those of Mn_3_O_4_,^11−13^ NiO,^58^ and ZnMn_2_O_4_.^14^ NaNiO_2_ also displays a much smaller magnitude of  than LaMnO_3_, with LaMnO_3_ shifting *T*_JT_ from ∼750 K at ambient pressure to room temperature in 12 GPa^2^ compared with a very small shift from ∼480 K at ambient pressure in NaNiO_2_.

Further variable-pressure diffraction measurements, at several temperatures and higher pressures, are needed to fully understand the process of suppressing the JT distortion in NaNiO_2_. Variable-pressure Raman spectroscopy measurements on NaNiO_2_ could also be useful and may help identify phase transitions at higher pressures.

Additionally, future investigations are needed to investigate whether other JT-distorted materials exhibit a d*T*_JT_/d*P* > 0 pressure dependence, for example, a study building on previous work on CuMnO_2_^16^ by measuring at multiple isotherms.

## Supporting Information Available

Additional neutron diffraction data and refinement details, SEM images, tables of diffraction data and distortion parameters, and transformation matrices (PDF)

## Notes

The variable-pressure neutron diffraction data from the PEARL instrument at ISIS are available at doi:10.5286/ISIS.E.RB2000219.^59^ All other data can be found at doi:10.17863/CAM.81605.^60^
