# Supplementary material for: Pressure Tuning the Jahn–Teller Transition Temperature in NaNiO2
Source: Inorg Chem. 2022 Mar 3;61(10):4312–21. doi: 10.1021/acs.inorgchem.1c03345 (PMC9098164; doi:10.1021/acs.inorgchem.1c03345)
Supplement: Supplementary file 1 — ic1c03345_si_001.pdf [file ic1c03345_si_001.pdf]

SUPPLEMENTARY MATERIAL: Pressure tuning the Jahn-Teller transition temperature in $NaNiO_2$

Liam A. V. Nagle-Cocco,*,† Craig L. Bull,‡,¶ Christopher J. Ridley,‡ and Siân E. Dutton*,†

†*Cavendish Laboratory, University of Cambridge, JJ Thomson Avenue, Cambridge, CB3 0HE, United Kingdom.*

‡*ISIS Neutron and Muon Facility, Rutherford Appleton Laboratory, Didcot, OX11 0QX, United Kingdom.*

¶*School of Chemistry, University of Edinburgh, David Brewster Road, Edinburgh, EH9 3FJ, United Kingdom.*

E-mail: lavn2@cam.ac.uk; sed33@cam.ac.uk

Contents

List of Figures **S-3**

List of Tables **S-3**

1 Relating neutron Time-of-Flight to d-spacing **S-5**

2 Peakshape used in neutron data analysis **S-5**

3 Reaction of the sample with pressure medium at elevated temperature **S-5**

4 Emergence of peaks in the sample-holder on PEARL **S-6**

5 Birch-Murnaghan Equation of State **S-6**

6 Room-temperature X-ray diffraction **S-7**

7 Polyhedral distortion parameters **S-7**

7.1 Bond length distortion index S-7

7.2 Effective coordination S-8

7.3 Van Vleck Q_2 distortion mode S-9

8 Bond Valence Sum parameters **S-9**

9 Transformation matrix between $C2/m$ and $R\bar{3}m$ space groups **S-10**

References **S-34**

List of Figures

S1 Rietveld refinement of room temperature XRD S-11

S2 Neutron diffraction data showing reaction of $NaNiO_2$ with pressure medium . S-12

S3 Neutron diffraction data for sample setup excluding $NaNiO_2$ S-13

S4 Monoclinic⇒rhombohedral phase transition with easing of pressure at 490 K S-14

S5 Na-O bond lengths with pressure S-15

S6 Ni-O bond lengths with pressure S-16

S7 Dependence on pressure of unit cell component volumes S-17

S8 Octahedral distortion parameters with pressure for monoclinic $NaNiO_2$ . . . S-18

S9 Rhombohedral compared with average monoclinic Ni-O bond length S-19

S10 Octahedral bond angle distortions, Δ, with pressure S-20

S11 Visual representation of principal axes compared with crystallographic axes . S-21

S12 Bond valence parameter M_i with pressure at 290 K for NaO_6 and NiO_6 octahedraS-22

S13 SEM images of $NaNiO_2$ powder S-23

S14 Diagram explaining the Δ angular distortion parameter S-24

List of Tables

S1 Lattice parameters from Rietveld refinement of room temperature XRD . . . S-23

S2 Lattice parameters of $NaNiO_2$ at ambient pressure and variable temperature S-25

S3 Lattice parameters of $NaNiO_2$ at 290 K and variable pressure S-26

S4 Lattice parameters of $NaNiO_2$ at 460 K and variable pressure S-27

S5 Lattice parameters of monoclinic $NaNiO_2$ at 490 K and variable pressure . . S-28

S6 Lattice parameters of rhombohedral $NaNiO_2$ at 490 K and variable pressure . S-29

S7 Distortion parameters for octahedra in $NaNiO_2$ at ambient pressure S-30

S8 Distortion parameters for octahedra in $NaNiO_2$ at 290 K S-31

S9 Distortion parameters for octahedra in $NaNiO_2$ at 460 K S-31

S10 Distortion parameters for octahedra in $NaNiO_2$ at 490 K S-32
S11 Matrix transformations from unit cell axes to principal axes for $NaNiO_2$ . . . S-33
S12 Parameters obtained from Birch-Murnaghan equation of state fits for $NaNiO_2$ S-33

1 Relating neutron Time-of-Flight to d-spacing

The relation between neutron Time-of-Flight (ToF) and the crystallographic d is given by a linear and quadratic term, along with a constant offset. The quadratic coefficient is always refined as a free parameter. The linear term and constant offset are usually fixed to values obtained from a standard NIST Si-640e/NAC data set, with the exceptions of bank 2 data from NOMAD where the constant offset is refined owing to the geometry of the forward scattering frames, and the linear term is refined on banks 2, 3, and 4.

2 Peakshape used in neutron data analysis

For the neutron data from PEARL, the built-in TOPAS 5 macro TOF_PV was used.[1,2] Different peakshape functions were used for the data from NOMAD. For the low-resolution frames (bank 2 and 3), a back-to-back exponential function convoluted with a symmetrical Pseudo-Voigt (TOF-PV) function[2] was used to describe the peak profile. For the high-resolution frames (bank 4 and 5), the back-to-back exponential function was replaced by a modified Ikeda-Carpenter-David function[3] to account for the strong peak asymmetry from moderator induced line profile.

3 Reaction of the sample with pressure medium at elevated temperature

Before any of the measurements presented in this paper were taken, a preliminary variable-temperature measurement was taken with $NaNiO_2$ in the sample-holder with a force of 7 Tonnes applied (<0.5 GPa) with a pressure medium of deuterated methanol:ethanol (4:1 by volume). Figure S2 shows that after heating to $\sim$470 K and cooling, the sample had undergone irreversible changes, likely due to reaction with the -OH groups in the pressure medium. For this reason, all subsequent measurements on PEARL at high temperatures

(460 K and 490 K) were performed with fluorinert as the pressure medium.

4 Emergence of peaks in the sample-holder on PEARL

In the 460 K and 490 K variable-pressure isotherms, neutron diffraction on PEARL showed the emergence of new peaks. Measurement of the sample setup without $NaNiO_2$ [Figure S3] confirmed that these peaks are not due to the $NaNiO_2$ but rather to one of the other phases present. Given that fluorinert is not perfectly hydrostatic at high pressures[4] and was present only at these two temperatures and not for the 290 K, we suggest these peaks may be attributable to fluorinert.

5 Birch-Murnaghan Equation of State

The second-order and third-order Birch-Murnaghan equations of state[5] used in this study [Table S12] are, respectively:

$$p(V) = \frac{3B}{2}(\eta^7 - \eta^5), \tag{1}$$

and;

$$p(V) = \frac{3B_0}{2}(\eta^7 - \eta^5)\left[1 + \frac{3}{4}(B' - 4)(\eta^2 - 1)\right], \tag{2}$$

where B is the bulk modulus, B_0 is the bulk modulus at zero pressure, B' is the derivative of bulk modulus with pressure, and the value of η is given by:

$$\eta = \left[\frac{V_0}{V}\right]^{1/3} \tag{3}$$

in which V_0 is the zero-pressure unit-cell volume. In the second-order Birch-Murnaghan equation of state, B' is fixed at 4. For all fits using a 3rd-order fit, B' was found to be close

to 4, and so the 2nd-order equation is used in the manuscript. The value of bulk modulus obtained from the 2nd-order fit was always within 3σ of that obtained by the 3rd-order fit.

6 Room-temperature X-ray diffraction

Initial XRD was performed to check the sample was consistent with literature reports. SI Table S1 shows the lattice parameters we obtained, which are consistent with previous literature reports (such as Refs.[6,7]). The Rietveld refinement is shown in Figure S1. XRD peaks were modelled using a Thompson-Cox-Hastings Pseudo-Voigt peak-shape.[8]

7 Polyhedral distortion parameters

Figure S8 shows the bond length distortion index[9] and effective coordination[10] for the NiO_6 and NaO_6 octahedra in monoclinic and rhombohedral $NaNiO_2$ respectively. These parameters are calculated using custom-written code for TOPAS 5.[1] This section defines these parameters, along with the Van Vleck Q_2 distortion mode.[11]

7.1 Bond length distortion index

The bond length distortion index, D, for a polyhedron with a coordination number n (i.e. 6 for octahedra), is defined as:

$$D = \frac{1}{n}\sum_{i=1}^{n}\frac{|l_i - l_{\text{av}}|}{l_{\text{av}}} \tag{4}$$

where l_i is the distance between the core cation and the ith coordinated anion, and l_{av} is the average of all the distances between the core cation and coordinated anions.

For octahedra elongated in one axis parallel to M-O (M=Na,Ni) bonds, such as the NiO_6 and NaO_6 octahedra in monoclinic $NaNiO_2$, this equation becomes in practice:

$$D = \frac{1}{3}\frac{l_{\text{long}} - l_{\text{av}}}{l_{\text{av}}} + \frac{2}{3}\frac{l_{\text{av}} - l_{\text{short}}}{l_{\text{av}}} \tag{5}$$

where l_{long} and l_{short} are the long and short bonds respectively.

7.2 Effective coordination

Effective coordination, ECoN, is a measure of the coordination where atoms are given a weighting based on their distance from the core cation relative to the average cation-anion distance, and is defined as:

$$\text{ECoN} = \sum_{i=1}^{n} \exp\left[1 - \left(\frac{l_i}{l'_{\text{av}}}\right)^6\right] \tag{6}$$

Here, l'_{av} is not a normal mean average, but a weighted average bond length:

$$l'_{\text{av}} = \frac{\sum_{i=1}^{n} l_i \exp\left[1 - \left(\frac{l_i}{l_{\text{min}}}\right)^6\right]}{\sum_{i=1}^{n} \exp\left[1 - \left(\frac{l_i}{l_{\text{min}}}\right)^6\right]} \tag{7}$$

where l_{min} is the minimum core cation-anion bond length.

We again define ECoN and l'_{av} specifically for the NaO_6 and NiO_6 octahedra. We take l'_{av} as:

$$l'_{\text{av}} = \frac{l_{\text{long}} \exp\left[1 - \left(\frac{l_{\text{long}}}{l_{\text{short}}}\right)^6\right] + 2l_{\text{short}}}{\exp\left[1 - \left(\frac{l_{\text{long}}}{l_{\text{short}}}\right)^6\right] + 2} \tag{8}$$

and ECoN is:

$$\text{ECoN} = 4\exp\left[1 - \left(\frac{l_{\text{short}}}{l'_{\text{av}}}\right)^6\right] + 2\exp\left[1 - \left(\frac{l_{\text{long}}}{l'_{\text{av}}}\right)^6\right] \tag{9}$$

7.3 Van Vleck Q_2 distortion mode

An alternative metric for considering the effect of pressure on JT-distorted NiO_6 octahedra would be the Q_2 distortion mode proposed by Van Vleck.[11] Here we use the form of the mode as stated in Ref.[12] and given here:

$$Q_2 = \frac{l_{\text{long}} - l_{\text{short}}}{\sqrt{2}} \tag{10}$$

where l_{long} and l_{short} are the long and short Ni-O bond lengths, respectively.

In this work we have used effective coordination and bond length distortion index to describe the magnitude of JT distortion in $NaNiO_2$. However, it is common to find Q_2 used in the literature in reference to JT-distorted octahedra, and so for comparison to the literature Tables S7-S10 show values of this parameter at each pressure and temperature point. We have only performed this calculation for the monoclinic phase as for the rhombohedral phase Q_2 will always be zero. Similarly, we have only calculated for NiO_6 octahedra as the NaO_6 octahedra are not JT-distorted.

8 Bond Valence Sum parameters

The Bond Valence Sum (BVS) method[13] empirically relates the valence, V_0 (equivalent to formal oxidation state) of a cation with the bond lengths to surrounding anions. For each interaction between neighbouring ions, we can define the "bond valence", s, which is the number of electron pairs forming the bond. s can be calculated as follows:

$$s = \exp\left[\frac{R_0 - R}{B}\right] \tag{11}$$

where R is the length of the bond between neighbouring ions, and R_0 and B are empirical parameters for the specific anion and cation pair. In the main paper, we used a model which builds on the BVS method to relate the parameter M_i to octahedral compressibility.[14] We

define M_i generally here:

$$M_i = \sum_j \frac{R_{ij}}{B} s_i = \sum_j \frac{R_{ij}}{B} \exp\left[\frac{R_0 - R_{ij}}{B}\right] \tag{12}$$

for j anions surrounding a central cation. For an elongated octahedron such as NaO_6 or NiO_6 in monoclinic $NaNiO_2$ we define M_{4+2} as follows:

$$M_{4+2} = 4\frac{R_{\text{short}}}{B} \exp\left[\frac{R_0 - R_{\text{short}}}{B}\right] + 2\frac{R_{\text{long}}}{B} \exp\left[\frac{R_0 - R_{\text{long}}}{B}\right] \tag{13}$$

For Ni^{3+}-O^{2-} bonds we have used the empirical parameters $R_0 = 1.75\,\text{Å}$ and $B = 0.37\text{Å}$ and for Na^{+}-O^{2-} bonds we have used the empirical parameters $R_0 = 1.695\,\text{Å}$ and $B = 0.42\text{Å}$.[15]

9 Transformation matrix between $C2/m$ and $R\bar{3}m$ space groups

Table S11 gives the transformational matrices between the rhombohedral $R\bar{3}m$ and monoclinic $C2/m$ phases of $NaNiO_2$ with the principal axes of compression. To aid interpretation of these matrices, we present the transformation matrix from the lattice parameters for $C2/m$, (a_1, b_1, c_1) to $R\bar{3}m$ (a_2, b_2, c_2):

$$\begin{pmatrix} -\frac{1}{3} & -1 & 0 \\ \frac{1}{3} & -1 & 0 \\ -\frac{2}{3} & 0 & 1 \end{pmatrix} \begin{pmatrix} a_2 \\ b_2 \\ c_2 \end{pmatrix} = \begin{pmatrix} a_1 \\ b_1 \\ c_1 \end{pmatrix} \tag{14}$$

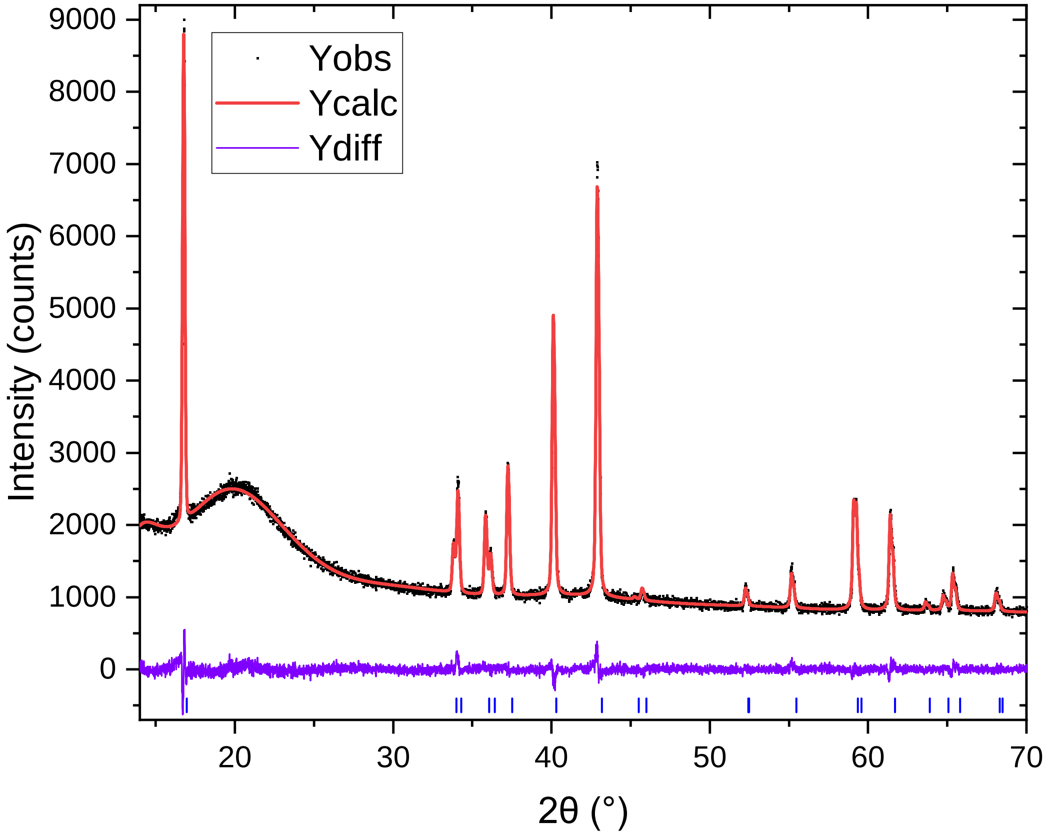

Figure S1: Room temperature powder XRD pattern for $NaNiO_2$, with Rietveld refinement.[16] Black dots: experimental data; red line: calculated pattern; purple line: difference pattern between the experimental and calculated pattern; blue tickmarks: Bragg reflection positions. Note that the broad peak above 20° is from the air-free sample holder.

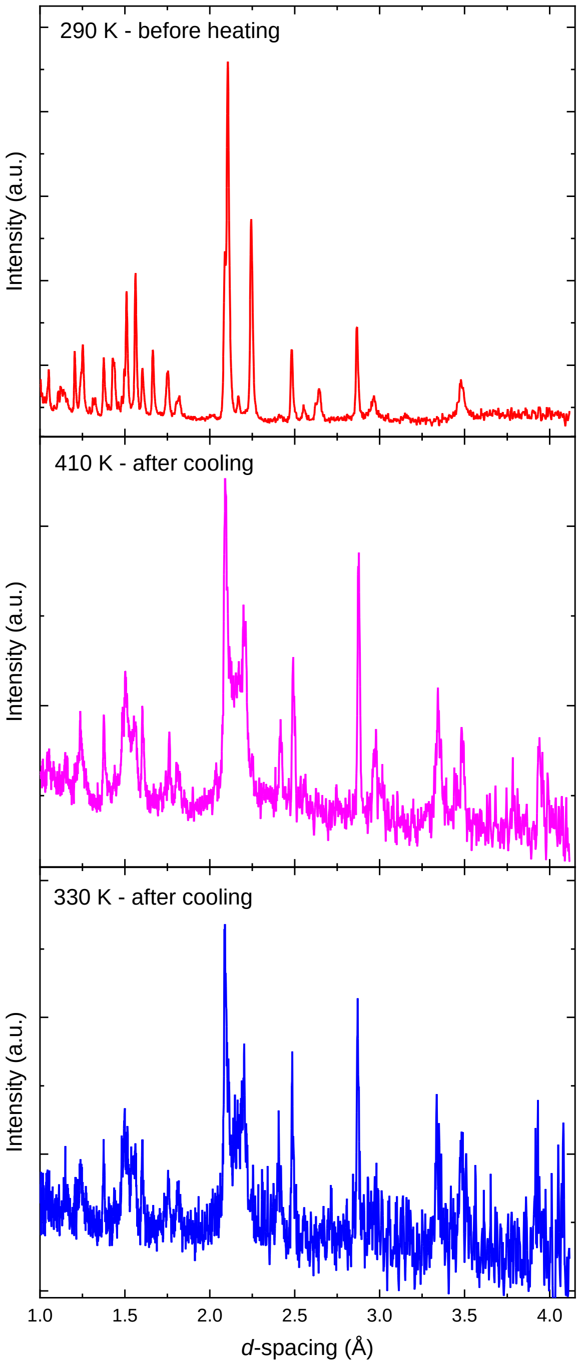

Figure S2: Neutron diffraction data from PEARL, under 7 Tonnes of force, with isobaric heating/cooling at 290 K (top), 410 K (middle), and 330 K (bottom). The pressure medium was deuterated methanol:ethanol solution. The structure of Bragg diffraction peaks exhibited irreversible changes during the heating/cooling process.

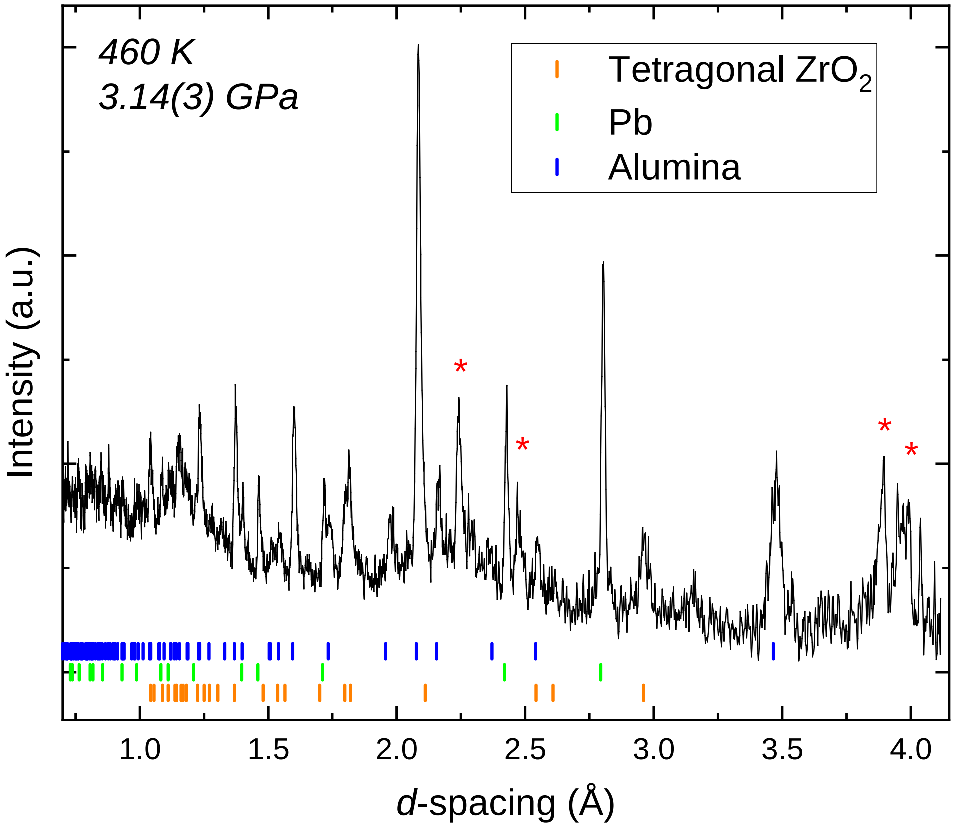

Figure S3: Representative neutron diffraction data from PEARL at ISIS, for the sample setup excluding $NaNiO_2$. Featured is the sample-holder, with fluorinert as the pressure medium. Red asterisks indicate peaks which emerged with heat and pressure, and which could not be fit with any known phases. These are attributed to a high temperature, high pressure crystalline phase of fluorinert.

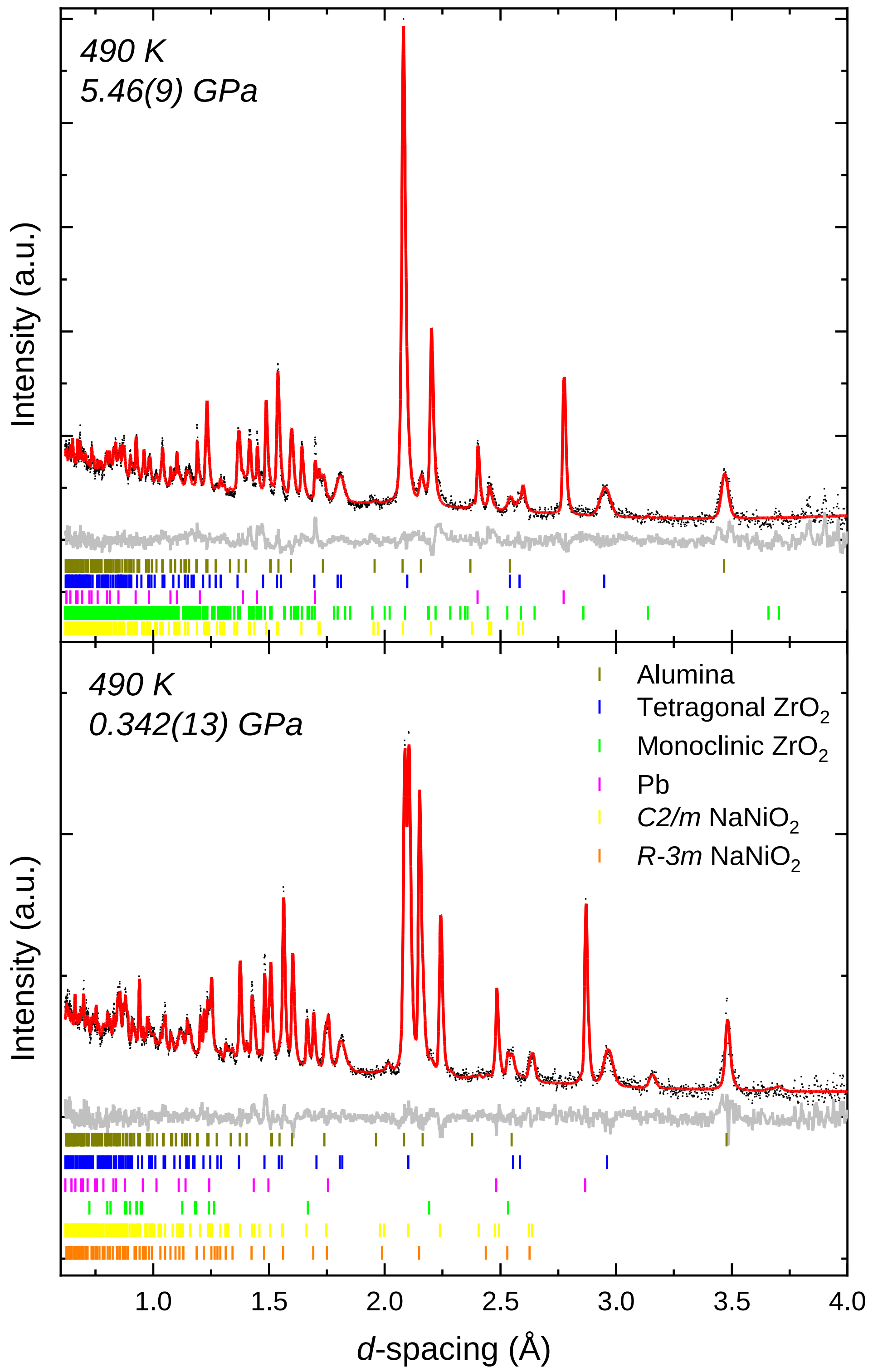

Figure S4: Neutron diffraction data, with Rietveld refinement, at 490 K at maximum pressure (top) and after easing off the pressure (bottom). This was performed following heating from 460 K to 490 K at the end of the variable-pressure 460 K isotherm.

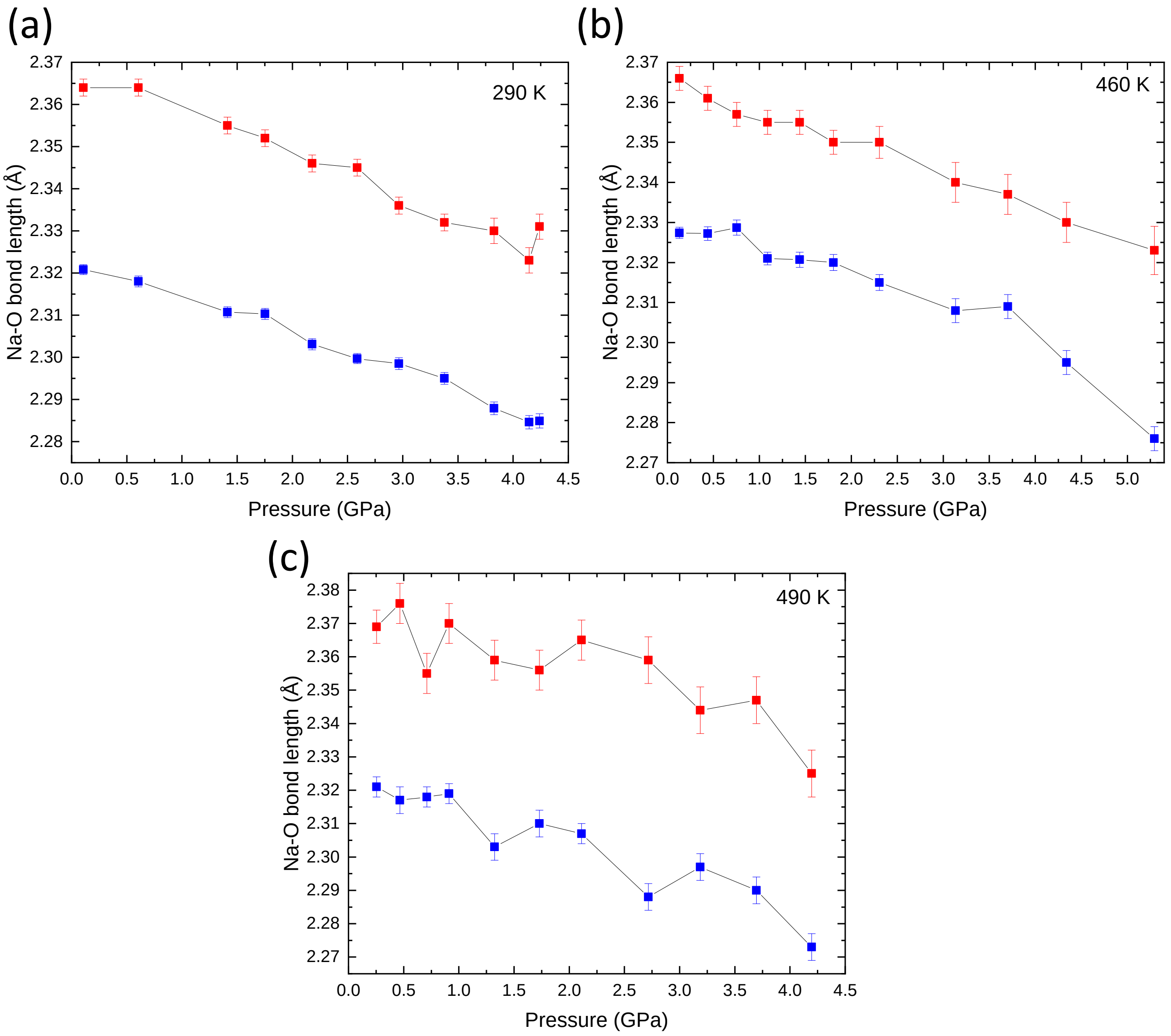

Figure S5: Dependence of Na-O bond length in monoclinic $C2/m$ $NaNiO_2$ with pressure at (a) 290 K, (b) 460 K, and (c) 490 K. Lines are a guide to the eye.

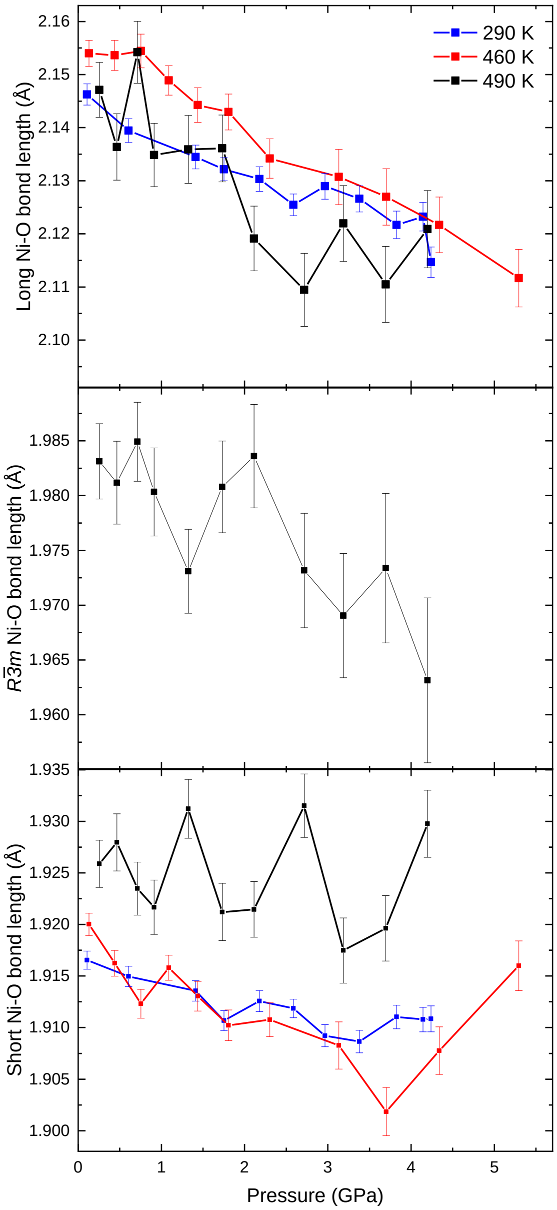

Figure S6: Dependence of Ni-O bond length in $NaNiO_2$ on pressure, for each of the long, short, and undistorted bonds. The long and short bonds are present at all three temperature points. The undistorted, $R\bar{3}m$ bonds are present only at 490 K where the rhombohedral phase is present. Lines are a guide to the eye.

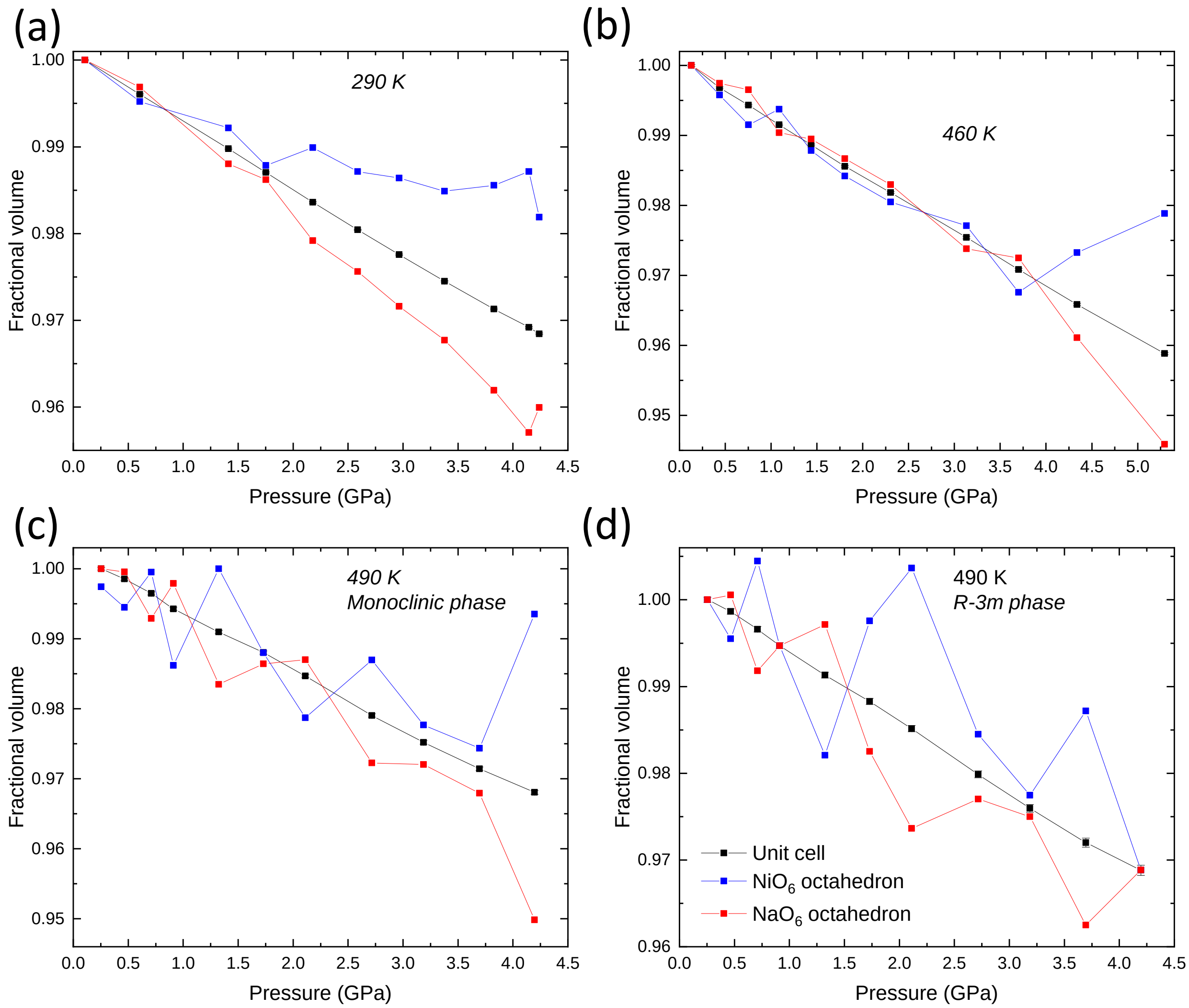

Figure S7: Dependence of unit cell volume, NiO_6 octahedral volume, and NaO_6 octahedral volume, on pressure, normalised to the starting values. (a) 290 K, (b) 460 K, and (c) and (d) 490 K. (c) and (d) represent the monoclinic and rhombohedral phases, respectively. Lines are a guide to the eye. Octahedral volumes are obtained using VESTA.[17] Errors are not calculated.

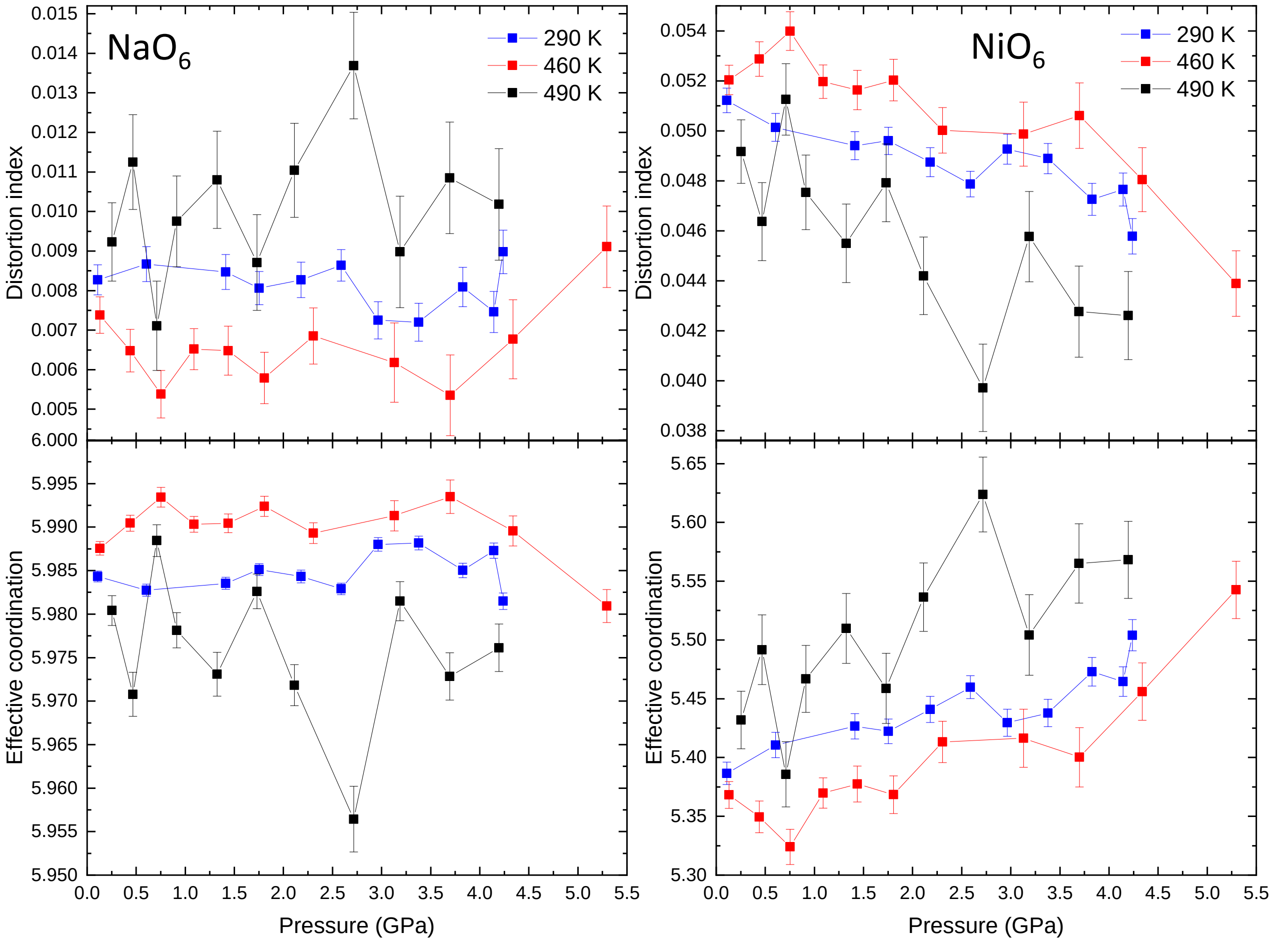

Figure S8: For monoclinic $C2/m$ $NaNiO_2$, from top to bottom: bond length distortion index and effective coordination number as a function of pressure at 290 K, 460 K, 490 K. Left is NaO_6 octahedra and right is NiO_6 octahedra. Lines are a guide to the eye.

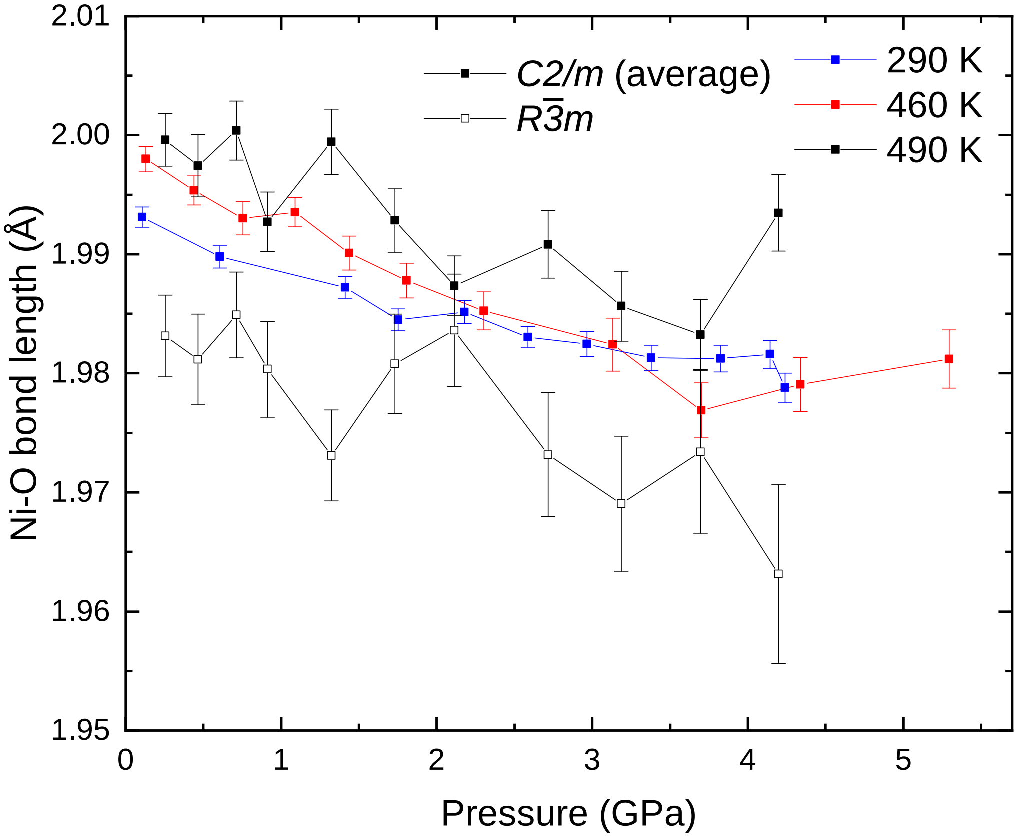

Figure S9: Ni-O bond lengths within octahedra for rhombohedral $NaNiO_2$ at 490 K, compared with the average bond lengths for JT-distorted monoclinic $NaNiO_2$ at 290 K, 460 K, and 490 K.

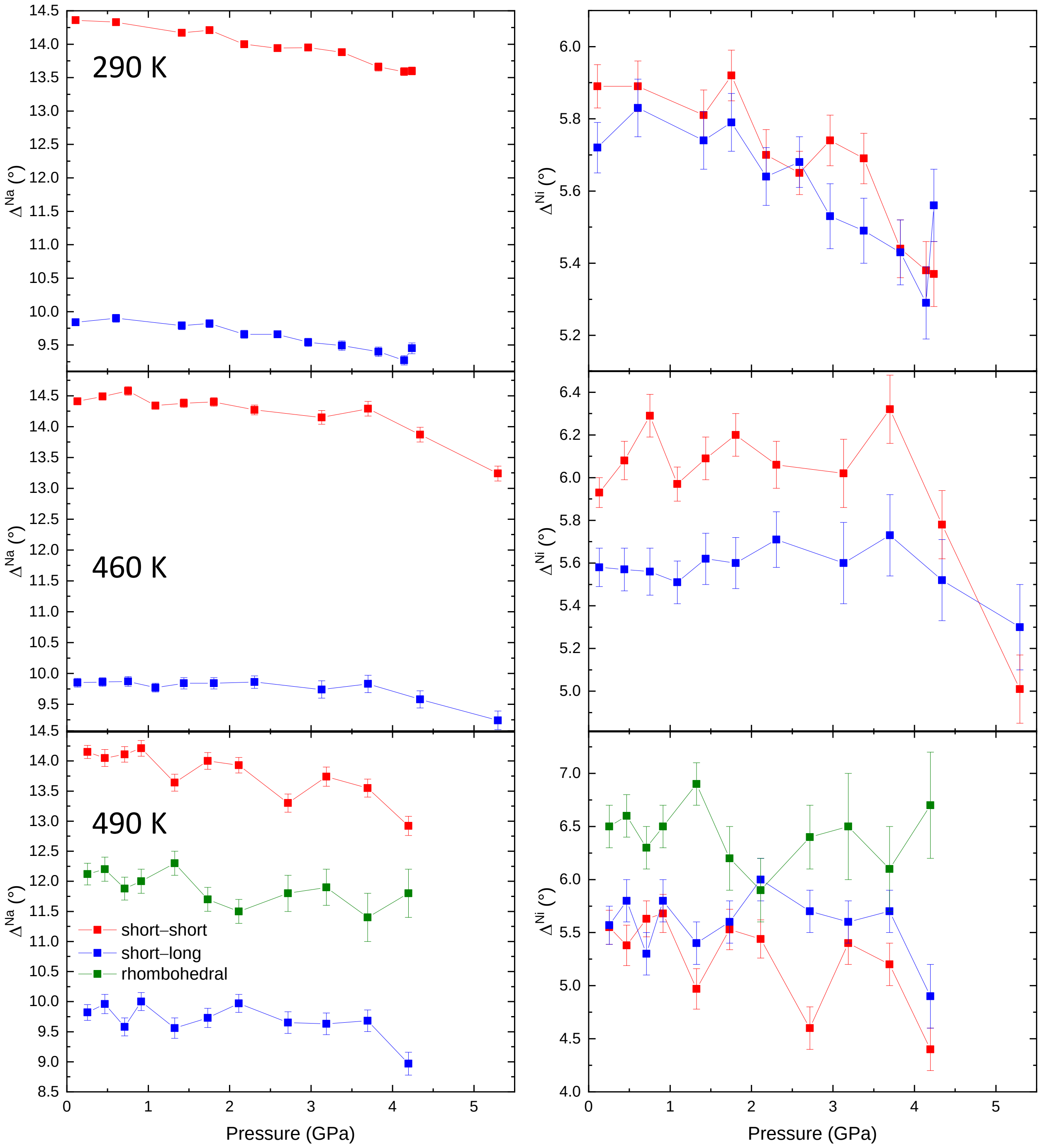

Figure S10: Octahedral bond angle distortion, Δ, as a function of pressure, for NiO_6 and NaO_6 octahedra with pressure for the monoclinic (290 K, 460 K, and 490 K) and rhombohedral (490 K only) phases of $NaNiO_2$. For monoclinic octahedra there are two Δ values as the angle between long and short bonds is distinct from the angle between short bonds. Lines are a guide to the eye.

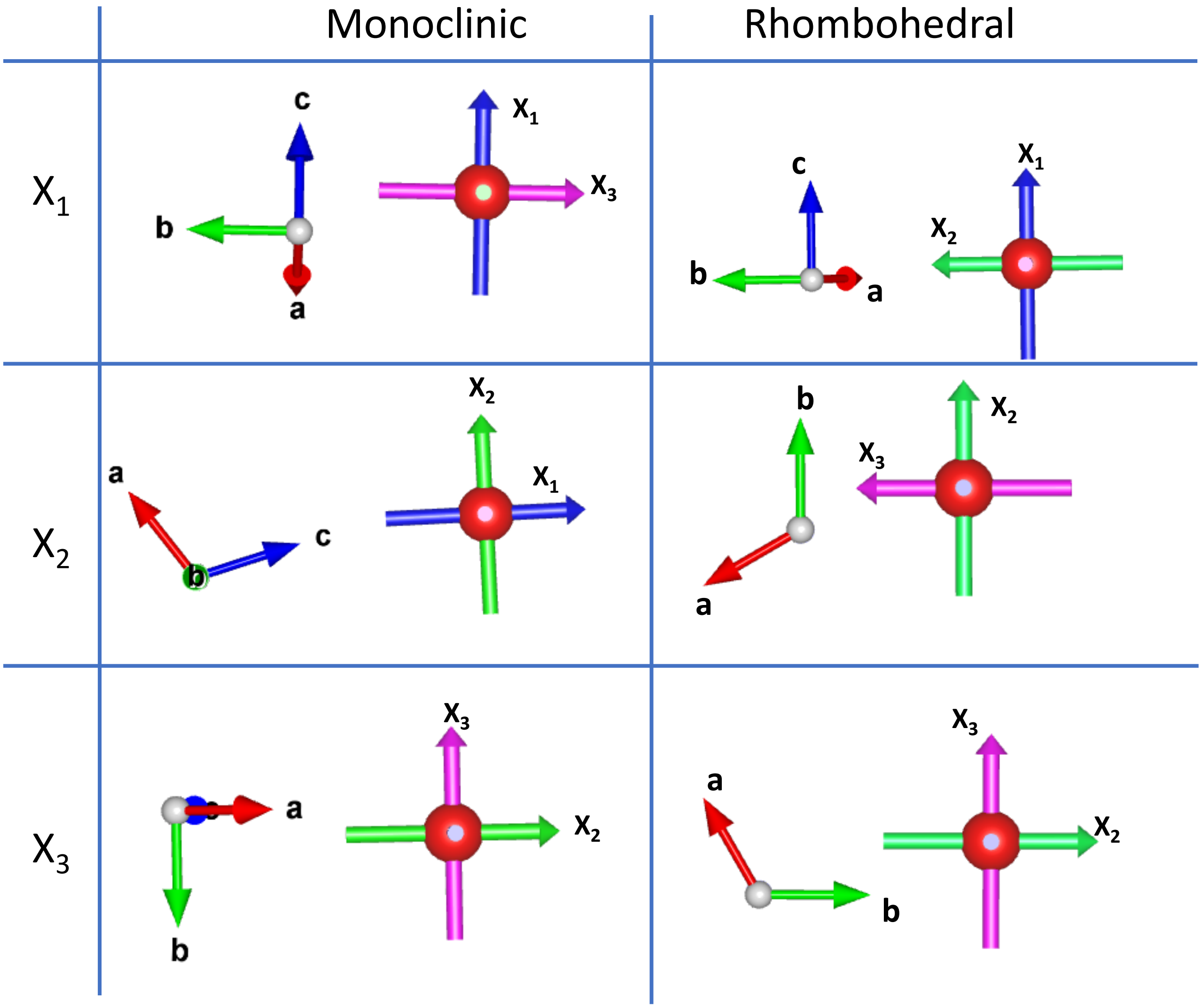

Figure S11: Visual representation of principal axes compared with crystallographic orientations. The transformation matrices are given in Table S11. This figure is made using VESTA.[17]

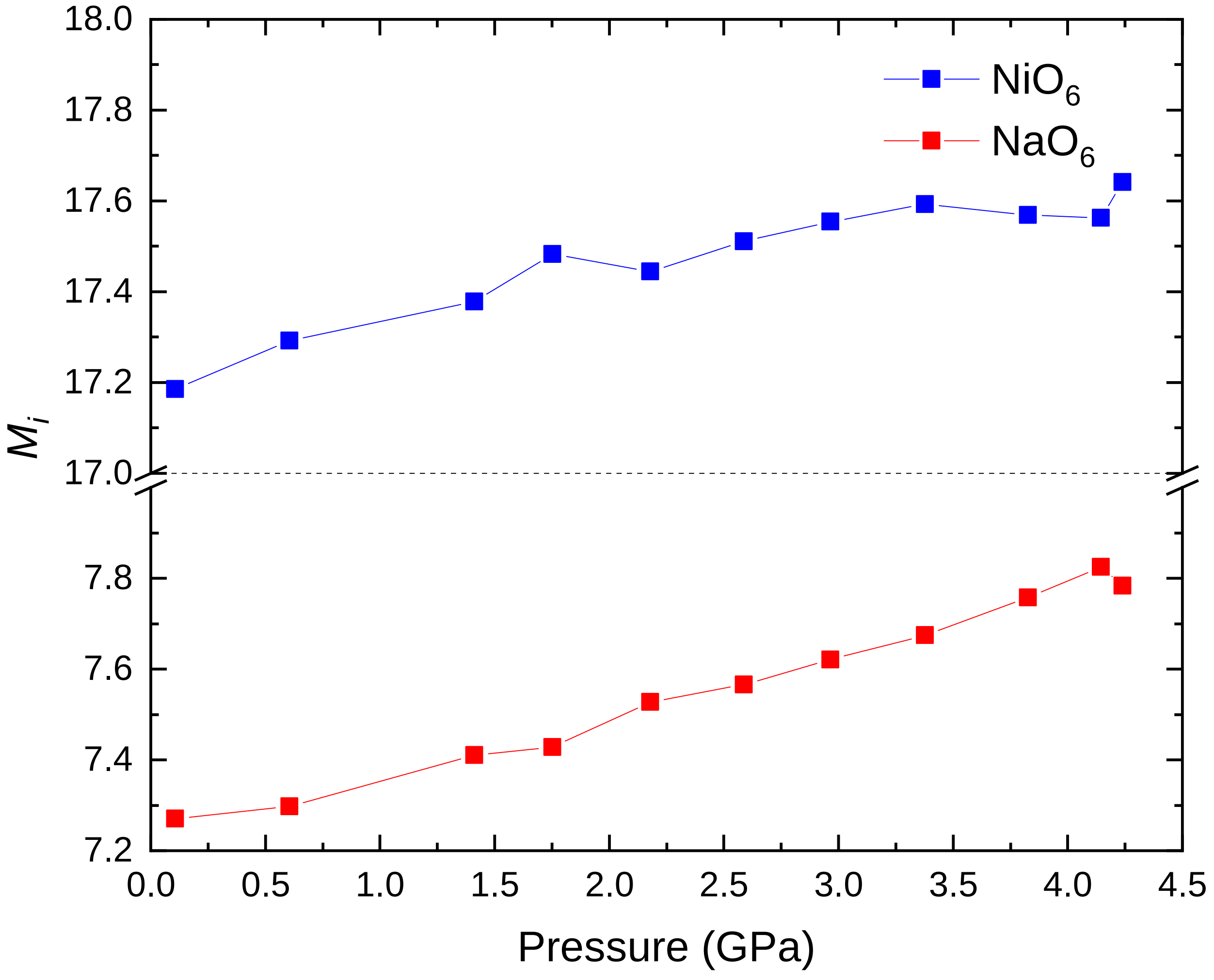

Figure S12: M_i, as defined in Eq. 13, with pressure at 290 K for NaO_6 and NiO_6 octahedra. Errors have not been determined.

(a) (b)

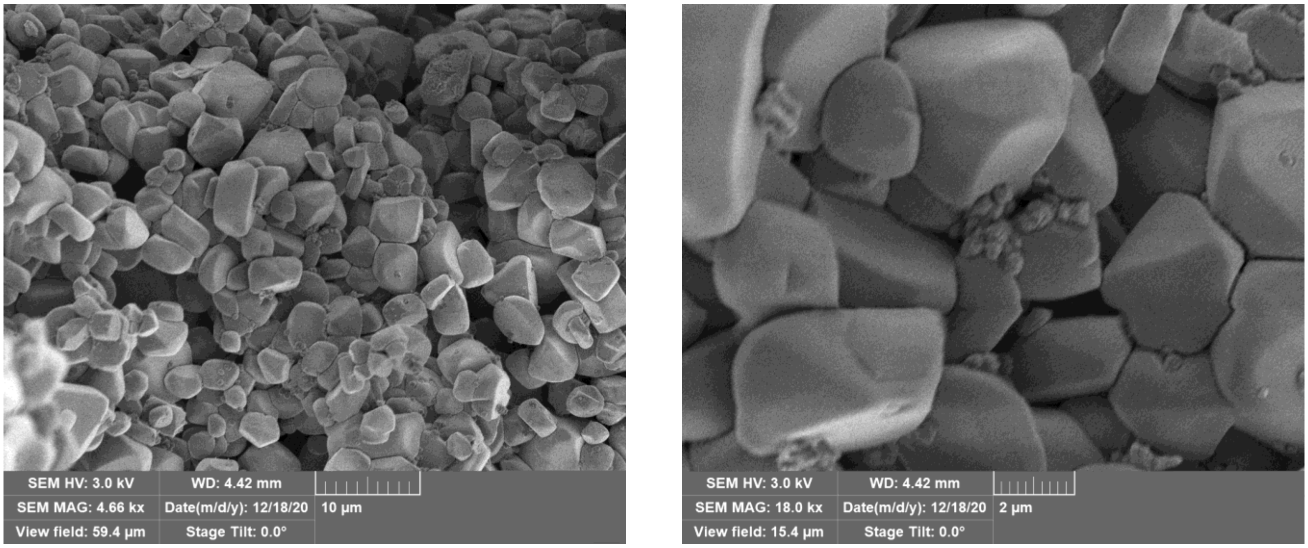

Figure S13: Images of $NaNiO_2$ taken using a Scanning Electron Microscope (SEM) at varying magnifications, with a view field of (a) 59.4 μm and (b) 15.4 μm.

Table S1: Lattice parameters of $NaNiO_2$ obtained by Rietveld refinement of the ambient-pressure, room-temperature XRD data.

a (Å)	b (Å)	c (Å)	β (°)
5.32202(10)	2.84711(5)	5.58439(13)	110.4676(13)

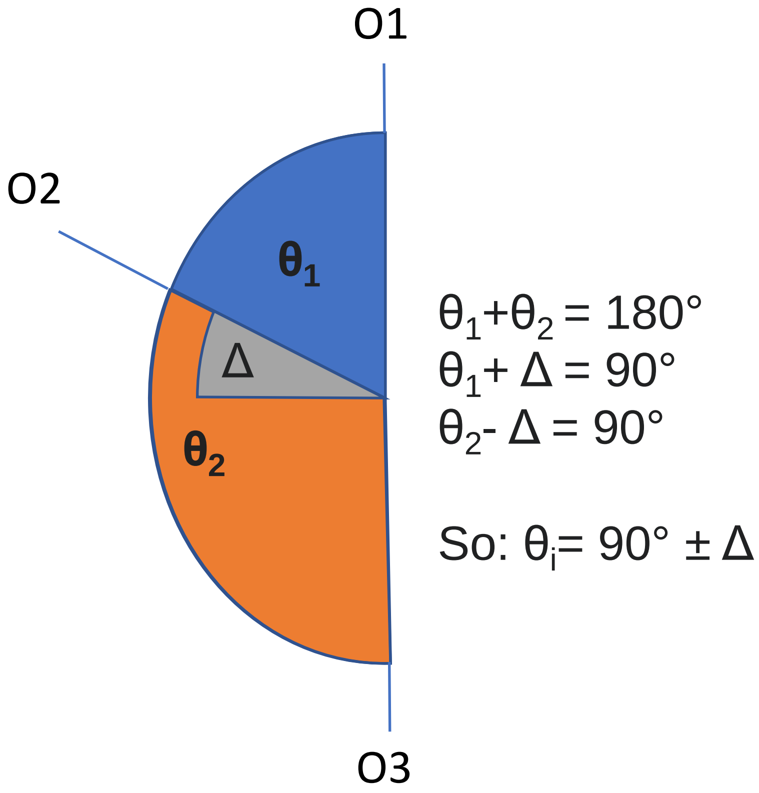

Figure S14: Half of the cross-section of an MO_6 (M=Na,Ni) octahedron in $NaNiO_2$, showing the origin of the Δ parameter defined in the manuscript. O1 and O3 are oxygen anions either side of a central cation, M, where the angle $\theta_{\mathrm{O1}-M-\mathrm{O3}} = 180°$. Given this constraint, the bond angles $\theta_1 = \theta_{\mathrm{O1}-M-\mathrm{O2}}$ and $\theta_2 = \theta_{\mathrm{O2}-M-\mathrm{O3}}$ can be described using a single parameter, which is their deviation from 90° which would be their angle in an undistorted octahedron.

Phase	T (K)	a (Å)	b (Å)	c (Å)	β (°)	O_x	O_z	B_{eq} Na	Ni	O
$C2/m$	293	5.31879(8)	2.84531(4)	5.58221(8)	110.4557(9)	0.28484(9)	0.80379(9)	0.55(2)	0.133(11)	0.266(14)
$C2/m$	450	5.32825(9)	2.85196(5)	5.59905(9)	110.4288(10)	0.28476(11)	0.80424(10)	1.00(14)	0.290(9)	0.45(17)
$R\bar{3}m$	500	2.96318(3)	-	15.77209(18)	-	-	0.23131(3)	1.219(13)	0.357(5)	0.981(8)
$C2/m$	316	5.32136(19)	2.846077(5)0	5.58536(9)	110.4530(10)	0.28479(10)	0.80383(9)	0.83(13)	0.157(12)	0.41(2)

Table S2: Lattice parameters of $NaNiO_2$ obtained by Rietveld refinement of the ambient-pressure, variable-temperature neutron diffraction data from NOMAD at the SNS. Uncertainties are shown in brackets. Site occupancy was fixed at 1. For the $C2/m$ phase, Ni, Na, and O ions occupy the $3b(0,0,\frac{1}{2})$, $3a(0,0,0)$, and $6c(0,0,z)$ Wyckoff sites respectively, and for the $R\bar{3}m$ phase, Ni, Na, and O ions occupy the $3b(0,0,\frac{1}{2})$, $3a(0,0,0)$, and $6c(0,0,z)$ Wyckoff sites respectively.

P (GPa)	a (Å)	b (Å)	c (Å)	β (°)	O_x	O_z	V ($Å^3$)	R_{wp}	R_p
0.107(8)	5.3191(3)	2.84585(13)	5.5820(2)	110.442(2)	0.2828(3)	0.8043(3)	79.195(6)	2.93141967	2.43586854
0.605(9)	5.3109(3)	2.8434(14)	5.5727(3)	110.422(3)	0.2823(4)	0.8045(4)	78.895(6)	3.23580481	2.66341279
1.411(11)	5.2978(3)	2.8397(15)	5.5575(3)	110.400(3)	0.2819(4)	0.8038(4)	78.377(6)	3.07798149	2.60253551
1.752(12)	5.2929(2)	2.83786(14)	5.5507(3)	110.389(3)	0.2823(4)	0.8041(4)	78.155(6)	2.91542517	2.49313043
2.179(16)	5.2857(3)	2.83577(14)	5.5425(3)	110.378(3)	0.2815(4)	0.8030(4)	77.879(6)	3.00792307	2.58842151
2.59(2)	5.2795(2)	2.83367(14)	5.5347(3)	110.369(3)	0.2810(3)	0.8029(3)	77.622(5)	2.63755132	2.23656634
2.97(3)	5.2742(3)	2.83169(14)	5.5276(3)	110.355(3)	0.2819(4)	0.8025(4)	77.405(6)	3.10089973	2.71114041
3.38(3)	5.2682(3)	2.82971(14)	5.5200(3)	110.345(3)	0.2817(4)	0.8022(4)	77.170(6)	3.10893472	2.69984789
3.83(4)	5.2618(3)	2.82769(15)	5.5123(3)	110.336(3)	0.2806(4)	0.8014(4)	76.914(7)	3.2119153	2.75615992
4.14(5)	5.2577(3)	2.82629(15)	5.5068(3)	110.326(3)	0.2807(4)	0.8007(4)	76.728(7)	3.27700635	2.8274854
4.24(5)	5.2565(3)	2.82571(16)	5.5050(3)	110.326(3)	0.2797(5)	0.8016(5)	76.704(7)	3.45422159	3.01386692

Table S3: Lattice parameters of monoclinic $C2/m$ $NaNiO_2$ obtained by Rietveld refinement of the variable-pressure neutron diffraction data at 290 K, from PEARL at ISIS. Uncertainties are shown in brackets. Site occupancy was fixed at 1. Ni, Na, and O ions occupy the $2a(0,0,0)$, $2d(0,\frac{1}{2},\frac{1}{2})$, and $4i(x,0,z)$ Wyckoff sites respectively.

P (GPa)	a (Å)	b (Å)	c (Å)	β (°)	O_x	O_z	V (Å^3)	R_{wp}	R_p
0.130(10)	5.3272(3)	2.8523(2)	5.5985(4)	110.421(3)	0.2833(4)	0.8040(4)	79.733(7)	3.30788518	2.84936867
0.438(11)	5.3211(4)	2.8498(2)	5.5914(4)	110.408(4)	0.2841(4)	0.8042(5)	79.476(8)	3.75748523	3.31752518
0.753(12)	5.3157(4)	2.8485(2)	5.5853(4)	110.395(4)	0.2851(5)	0.8046(5)	79.299(8)	4.14002653	3.69579066
1.089(11)	5.3102(4)	2.8467(2)	5.5781(4)	110.378(4)	0.2836(4)	0.8036(5)	79.072(7)	3.51511944	3.09627876
1.437(13)	5.3043(4)	2.8451(2)	5.5710(4)	110.369(4)	0.2837(5)	0.8042(5)	78.844(8)	4.09600581	3.64379456
1.806(16)	5.2976(4)	2.8434(2)	5.5634(4)	110.354(4)	0.2841(5)	0.8043(6)	78.592(9)	4.23441754	3.77667381
2.30(2)	5.2900(4)	2.8412(2)	5.5539(4)	110.332(4)	0.2830(6)	0.8043(6)	78.297(9)	4.49147271	3.59750423
3.13(3)	5.2769(5)	2.8371(3)	5.5384(5)	110.308(6)	0.2830(8)	0.8036(8)	77.747(13)	5.59089319	4.68593156
3.70(4)	5.2684(5)	2.8341(3)	5.5269(6)	110.300(6)	0.2839(8)	0.8046(9)	77.386(14)	5.57533367	4.74473122
4.34(6)	5.2588(5)	2.8306(3)	5.5151(5)	110.294(5)	0.2818(8)	0.8026(9)	77.010(13)	5.18154002	4.56558192
5.29(8)	5.2455(5)	2.8257(3)	5.4976(5)	110.270(6)	0.2784(8)	0.7998(9)	76.449(13)	4.92128375	4.13040048

Table S4: Lattice parameters of monoclinic $C2/m$ $NaNiO_2$ obtained by Rietveld refinement of the variable-pressure neutron diffraction data at 460 K, from PEARL at ISIS. Uncertainties are shown in brackets. Site occupancy was fixed at 1. Ni, Na, and O ions occupy the $3b(0,0,\frac{1}{2})$, $3a(0,0,0)$, and $6c(0,0,z)$ Wyckoff sites respectively.

P (GPa)	a (Å)	b (Å)	c (Å)	β (°)	O_x	O_z	V (Å^3)	R_{wp}	R_p
0.254(17)	5.3248(6)	2.8524(3)	5.5979(7)	110.407(6)	0.2814(8)	0.8031(8)	79.689(16)	6.64524073	5.73907058
0.47(2)	5.3212(7)	2.8516(4)	5.5953(8)	110.405(8)	0.2797(10)	0.8035(10)	79.573(19)	7.66167689	6.52440379
0.71(2)	5.3185(7)	2.8506(4)	5.5880(8)	110.392(7)	0.2826(9)	0.8021(9)	79.409(18)	7.17326338	6.17786741
0.91(2)	5.3133(7)	2.8489(4)	5.5835(8)	110.369(8)	0.2810(10)	0.8043(10)	79.232(19)	7.33515484	6.4207228
1.32(2)	5.3054(7)	2.8470(4)	5.5769(8)	110.364(8)	0.2788(10)	0.8009(10)	78.969(19)	7.42007316	6.48156932
1.73(3)	5.3007(7)	2.8449(4)	5.5685(8)	110.346(7)	0.2810(10)	0.8027(10)	78.735(19)	7.31579921	6.37537941
2.11(3)	5.2941(7)	2.8432(4)	5.5592(7)	110.322(7)	0.2791(10)	0.8039(10)	78.469(18)	7.72857288	6.78584151
2.72(4)	5.2829(7)	2.8396(4)	5.5452(8)	110.300(8)	0.2756(11)	0.8011(11)	78.019(20)	8.22707357	7.03627183
3.18(4)	5.2758(7)	2.8371(4)	5.5360(8)	110.304(8)	0.2800(12)	0.8022(11)	77.712(20)	8.2544413	7.10556935
3.70(5)	5.2676(8)	2.8346(5)	5.5274(9)	110.289(9)	0.2781(11)	0.8021(11)	77.411(21)	8.41755176	7.27371179
4.20(6)	5.2613(8)	2.8323(5)	5.5181(9)	110.259(9)	0.2769(11)	0.7975(12)	77.143(22)	8.37037449	7.31152852

Table S5: Lattice parameters of monoclinic $C2/m$ $NaNiO_2$ obtained by Rietveld refinement of the variable-pressure neutron diffraction data at 490 K, from PEARL at ISIS. Uncertainties are shown in brackets. Site occupancy was fixed at 1. Ni, Na, and O ions occupy the $3b(0,0,\frac{1}{2})$, $3a(0,0,0)$, and $6c(0,0,z)$ Wyckoff sites respectively.

Table S6: Lattice parameters, unit cell parameters, and fitting metrics for rhombohedral $R\bar{3}m$ $NaNiO_2$ obtained by Rietveld refinement of the variable-pressure neutron diffraction data at 490 K, from PEARL at ISIS. Uncertainties are shown in brackets. Site occupancy was fixed at 1. For the rhombohedral phase, Ni, Na, and O ions occupy the $3b(0,0,\frac{1}{2})$, $3a(0,0,0)$, and $6c(0,0,z)$ Wyckoff sites respectively.

P (GPa)	a (Å)	c (Å)	O_z	V (Å^3)	R_{wp}	R_p
0.254(17)	2.9591(3)	15.758(3)	0.2306(4)	119.497(35)	6.64524073	5.73907058
0.47(2)	2.9582(4)	15.747(3)	0.2304(5)	119.336(38)	7.66167689	6.52440379
0.71(2)	2.9565(4)	15.733(3)	0.2311(4)	119.092(37)	7.17326338	6.17786741
0.91(2)	2.9549(4)	15.720(3)	0.2306(5)	118.865(41)	7.33515484	6.4207228
1.32(2)	2.9522(4)	15.695(3)	0.2300(5)	118.459(40)	7.42007316	6.48156932
1.73(3)	2.9497(4)	15.673(4)	0.2312(5)	118.097(43)	7.31579921	6.37537941
2.11(3)	2.9467(4)	15.655(4)	0.2318(6)	117.722(44)	7.72857288	6.78584151
2.72(4)	2.9424(4)	15.617(4)	0.2309(6)	117.091(48)	8.22707357	7.03627183
3.18(4)	2.9391(5)	15.589(5)	0.2307(7)	116.623(54)	8.2544413	7.10556935
3.70(5)	2.9364(6)	15.555(6)	0.2316(8)	116.151(63)	8.41755176	7.27371179
4.20(6)	2.9345(6)	15.524(6)	0.2306(9)	115.771(70)	8.37037449	7.31152852

Space group	T (K)	Bond length distortion index		Effective coordination		Q_2 amplitude
		NaO_6	NiO_6	NaO_6	NiO_6	NiO_6
$C2/m$	293	0.00581(11)	0.05463(14)	5.99232(19)	5.309(3)	0.1751(6)
$C2/m$	450	0.00598(13)	0.05414(16)	5.9919(2)	5.321(3)	0.1723(5)
$R\bar{3}m$	500	**0**	**0**	**6**	**6**	**0**
$C2/m$	316	0.00591(12)	0.05455(16)	5.9921(2)	5.311(3)	0.1633(8)

Table S7: Bond length distortion index and effective coordination for NaO_6 and NiO_6 octahedra, and Q_2 amplitude of NiO_6 octahedra, in $NaNiO_2$ at ambient pressure, based on the oxygen positions obtained from Rietveld refinement of the neutron diffraction data from NOMAD. Values in **bold** are fixed by the symmetry of the space group.

Table S8: Bond length distortion index and effective coordination for NaO_6 and NiO_6 octahedra, and Q_2 amplitude of NiO_6 octahedra, in monoclinic $C2/m$ $NaNiO_2$ at 290 K for various pressures, based on the oxygen positions obtained from Rietveld refinement of the neutron diffraction data from PEARL.

P (GPa)	Bond length distortion index		Effective coordination		Q_2 amplitude
	NaO_6	NiO_6	NaO_6	NiO_6	NiO_6
0.107(8)	0.0083(4)	0.0512(5)	5.9843(6)	5.387(10)	0.1624(16)
0.605(9)	0.0087(4)	0.0501(6)	5.9828(7)	5.411(11)	0.1587(17)
1.411(11)	0.0085(4)	0.0494(6)	5.9835(7)	5.427(11)	0.1562(17)
1.752(12)	0.0081(4)	0.0496(6)	5.9851(7)	5.422(11)	0.1566(17)
2.179(16)	0.0083(5)	0.0488(6)	5.9843(7)	5.441(11)	0.1540(18)
2.59(2)	0.0086(4)	0.0479(5)	5.9829(7)	5.460(10)	0.1511(16)
2.97(3)	0.0073(5)	0.0493(6)	5.9880(8)	5.430(12)	0.1554(19)
3.38(3)	0.0072(5)	0.0489(6)	5.9882(8)	5.438(12)	0.1541(19)
3.83(4)	0.0081(5)	0.0473(6)	5.9850(8)	5.473(12)	0.149(2)
4.14(5)	0.0075(5)	0.0477(7)	5.9873(9)	5.465(13)	0.150(2)
4.24(5)	0.0090(5)	0.0458(7)	5.9815(10)	5.504(13)	0.144(2)

Table S9: Bond length distortion index and effective coordination for NaO_6 and NiO_6 octahedra, and Q_2 amplitude of NiO_6 octahedra, in monoclinic $C2/m$ $NaNiO_2$ at 460 K for various pressures, based on the oxygen positions obtained from Rietveld refinement of the neutron diffraction data from PEARL.

P (GPa)	Bond length distortion index		Effective coordination		Q_2 amplitude
	NaO_6	NiO_6	NaO_6	NiO_6	NiO_6
0.130(10)	0.0074(5)	0.0520(6)	5.9876(8)	5.368(11)	0.165(2)
0.438(11)	0.0065(5)	0.0529(7)	5.9904(9)	5.350(13)	0.168(2)
0.753(12)	0.0054(6)	0.0540(8)	5.9934(11)	5.324(15)	0.171(2)
1.089(11)	0.0065(5)	0.0520(7)	5.9903(9)	5.370(13)	0.165(2)
1.437(13)	0.0065(6)	0.0516(8)	5.9904(10)	5.377(15)	0.163(3)
1.806(16)	0.0058(7)	0.0520(8)	5.9924(12)	5.368(16)	0.165(3)
2.30(2)	0.0069(7)	0.0500(9)	5.9893(12)	5.413(18)	0.158(3)
3.13(3)	0.0062(10)	0.0499(13)	5.9913(18)	5.42(3)	0.157(4)
3.70(4)	0.0054(10)	0.0506(13)	5.9935(19)	5.40(3)	0.159(4)
4.34(6)	0.0068(10)	0.0481(13)	5.9896(17)	5.46(2)	0.151(4)
5.29(8)	0.0091(10)	0.0439(13)	5.9809(19)	5.54(2)	0.138(4)

P (GPa)	Bond length distortion index		Effective coordination		Q_2 amplitude
	NaO_6	NiO_6	NaO_6	NiO_6	NiO_6
0.254(17)	0.0092(10)	0.0492(13)	5.9804(17)	5.43(2)	0.156(4)
0.47(2)	0.0113(12)	0.0464(16)	5.971(3)	5.49(3)	0.147(5)
0.71(2)	0.0071(11)	0.0513(14)	5.9885(18)	5.39(3)	0.163(5)
0.91(2)	0.0098(12)	0.0475(15)	5.978(2)	5.47(3)	0.151(5)
1.32(2)	0.0108(12)	0.0455(16)	5.973(3)	5.51(3)	0.145(5)
1.73(3)	0.0087(12)	0.0479(16)	5.983(2)	5.46(3)	0.152(5)
2.11(3)	0.0110(12)	0.0442(16)	5.972(2)	5.54(3)	0.140(5)
2.72(4)	0.0137(14)	0.0397(18)	5.956(4)	5.62(3)	0.126(5)
3.18(4)	0.0090(14)	0.0458(18)	5.981(2)	5.50(3)	0.145(6)
3.70(5)	0.0109(14)	0.0428(18)	5.973(3)	5.57(3)	0.135(6)
4.20(6)	0.0102(14)	0.0426(18)	5.976(3)	5.57(3)	0.135(6)

Table S10: Bond length distortion index and effective coordination for NaO_6 and NiO_6 octahedra, and Q_2 amplitude of NiO_6 octahedra, in monoclinic $C2/m$ $NaNiO_2$ at 490 K for various pressures, based on the oxygen positions obtained from Rietveld refinement of the neutron diffraction data from PEARL. In the rhombohedral phase, bond length distortion index and effective coordination are constrained to 0 and 6 respectively by the symmetry of the space group.

Table S11: Axial compressibility, K, and non-normalised transformation matrices between the crystallographic axes a, b, and c, and the principal axes of compression, X_1, X_2, and X_3. This was obtained using PASCal.[18] These vectors are also displayed in Figure S11. When interpreting the directions of the vectors in the rhombohedral unit cell, note that within the ab-plane there is a C_6 rotational symmetry.

Phase	T (K)	Axis	$\vec{a}$	$\vec{b}$	$\vec{c}$	K (TPa^{-1})
$R\bar{3}m$	490	$\vec{X}_1$	0	0	1	3.69(10)
		$\vec{X}_2$	0.0002	1	0	2.23(12)
		$\vec{X}_3$	0.8945	0.4471	0	2.23(12)
$C2/m$	490	$\vec{X}_1$	-0.3700	0	0.9290	3.76(14)
		$\vec{X}_2$	0.8532	0	0.5215	2.82(12)
		$\vec{X}_3$	0	-1	0	1.849(2)
	460	$\vec{X}_1$	-0.4086	0	0.9127	3.53(2)
		$\vec{X}_2$	0.8415	0	0.5402	2.77(2)
		$\vec{X}_3$	0	-1	0	1.63(7)
	290	$\vec{X}_1$	-0.3180	0	0.9481	3.36(3)
		$\vec{X}_2$	0.8675	0	0.4974	2.62(4)
		$\vec{X}_3$	0	-1	0	1.76(3)

Table S12: Results of second- and third-order Birch-Murnaghan fitting (Eq. 1 and 2) for the three isotherms. For the second-order fits, B' is fixed at 4. These results were obtained using PASCal.[18]

Phase	T (K)	3rd-order V_0 (Å^3)	3rd-order B_0 (GPa)	3rd-order B'	2nd-order V_0 (Å^3)	2nd-order B (GPa)
$R\bar{3}m$	490	119.80(3)	117(4)	2(2)	119.83(2)	113(1)
$C2/m$	490	79.89(3)	112(5)	3(2)	79.900(16)	110(1)
	460	79.784(11)	117(2)	2.8(7)	79.798(9)	113.5(6)
	290	79.255(10)	121(2)	3.6(8)	79.258(7)	119.6(5)

References

(1) Coelho, A. A. TOPAS and TOPAS-Academic: an optimization program integrating computer algebra and crystallographic objects written in C++. *Journal of Applied Crystallography* **2018**, *51*, 210–218.

(2) Enzo, S.; Fagherazzi, G.; Benedetti, A.; Polizzi, S. A profile-fitting procedure for analysis of broadened X-ray diffraction peaks. I. Methodology. *Journal of Applied Crystallography* **1988**, *21*, 536–542.

(3) Ikeda, S.; Carpenter, J. M. Wide-energy-range, high-resolution measurements of neutron pulse shapes of polyethylene moderators. *Nuclear Instruments and Methods in Physics Research Section A: Accelerators, Spectrometers, Detectors and Associated Equipment* **1985**, *239*, 536–544.

(4) Sidorov, V.; Sadykov, R. Hydrostatic limits of Fluorinert liquids used for neutron and transport studies at high pressure. *Journal of Physics: Condensed Matter* **2005**, *17*, S3005.

(5) Birch, F. Finite elastic strain of cubic crystals. *Physical Review* **1947**, *71*, 809.

(6) Dick, S.; Müller, M.; Preissinger, F.; Zeiske, T. The structure of monoclinic $NaNiO_2$ as determined by powder X-ray and neutron scattering. *Powder Diffraction* **1997**, *12*, 239–241.

(7) Sofin, M.; Jansen, M. New route of preparation and properties of $NaNiO_2$. *Zeitschrift für Naturforschung B* **2005**, *60*, 701–704.

(8) Thompson, P.; Cox, D.; Hastings, J. Rietveld refinement of Debye-Scherrer synchrotron X-ray data from Al_2O_3. *Journal of Applied Crystallography* **1987**, *20*, 79–83.

(9) Baur, W. The geometry of polyhedral distortions. Predictive relationships for the phos-

phate group. *Acta Crystallographica Section B: Structural Crystallography and Crystal Chemistry* **1974**, *30*, 1195–1215.

(10) Hoppe, R. Effective coordination numbers (ECoN) and mean fictive ionic radii (MEFIR). *Zeitschrift für Kristallographie-Crystalline Materials* **1979**, *150*, 23–52.

(11) Van Vleck, J. The Jahn-Teller Effect and Crystalline Stark Splitting for Clusters of the Form XY_6. *The Journal of Chemical Physics* **1939**, *7*, 72–84.

(12) Schmitt, M. M.; Zhang, Y.; Mercy, A.; Ghosez, P. Electron-lattice interplay in $LaMnO_3$ from canonical Jahn-Teller distortion notations. *Physical Review B* **2020**, *101*, 214304.

(13) Brown, I.; Shannon, R. Empirical bond-strength–bond-length curves for oxides. *Acta Crystallographica Section A: Crystal Physics, Diffraction, Theoretical and General Crystallography* **1973**, *29*, 266–282.

(14) Zhao, J.; Ross, N. L.; Angel, R. J. New view of the high-pressure behaviour of $GdFeO_3$-type perovskites. *Acta Crystallographica Section B: Structural Science* **2004**, *60*, 263–271.

(15) Bond valence parameters (IUCr), *https://www.iucr.org/resources/data/datasets/bond-valence-parameters*. 2016 version. Accessed 27th September 2021. `https://www.iucr.org/resources/data/datasets/bond-valence-parameters`.

(16) Rietveld, H. A profile refinement method for nuclear and magnetic structures. *Journal of Applied Crystallography* **1969**, *2*, 65–71.

(17) Momma, K.; Izumi, F. VESTA 3 for three-dimensional visualization of crystal, volumetric and morphology data. *Journal of applied crystallography* **2011**, *44*, 1272–1276.

(18) Cliffe, M. J.; Goodwin, A. L. PASCal: a principal axis strain calculator for thermal expansion and compressibility determination. *Journal of Applied Crystallography* **2012**, *45*, 1321–1329.
